# Cochlear nucleus spatial transcriptomes of normal and hearing loss mice reveal a critical role of *Spp1* in bushy cells

**DOI:** 10.1038/s41422-026-01246-4

**Published:** 2026-04-06

**Authors:** Huihui Liu, Shangfeng Liao, Xiaowei Li, Li Song, Mu-Ming Poo, Jing Zhao, Weijun Zhou, Ruijie Cai, Meijian Wang, Xiaotong Ma, Shaohui Lin, Xingle Zhao, Ningyuan Zhu, Yuanwei Zhang, Junpu Mei, Lei Song, Lijian Zhao, Sidi Liu, Ying Chen, Hao Wu

**Affiliations:** 1 Department of Otolaryngology-Head and Neck Surgery, Shanghai Ninth People’s Hospital, Shanghai Jiao Tong University School of Medicine, Shanghai, China; 2 Ear Institute, Shanghai Jiao Tong University School of Medicine, Shanghai, China; 3 Shanghai Key Laboratory of Translational Medicine on Ear and Nose Diseases, Shanghai, China; 4 BGI Research, Shenzhen, Guangdong, China; 5 BGI Research, Sanya, Hainan, China; 6 Guangdong Cardiovascular Institute, Guangdong Provincial People’s Hospital, Guangdong Academy of Medical Sciences, Guangzhou, Guangdong, China; 7 Department of Pathology, Guangdong Provincial People’s Hospital (Guangdong Academy of Medical Sciences), Southern Medical University, Guangzhou, Guangdong, China; 8 Center for Excellence in Brain Science and Intelligence Technology, Institute of Neuroscience, Chinese Academy of Sciences, Shanghai, China; 9 Shanghai Key Laboratory of Psychotic Disorders, Shanghai Mental Health Center, Shanghai Jiao Tong University School of Medicine, Shanghai, China

**Keywords:** Transcriptomics, Mechanisms of disease

## Abstract

The molecular and cellular mechanisms underlying the function of the cochlear nucleus (CN) remain to be fully elucidated. Using single-nucleus RNA sequencing and single-cell spatial transcriptome analyses, we generated a comprehensive cell type atlas of the mouse CN, identified molecularly defined CN subregions, and quantified changes in gene expression and the spatial organization of CN cells in normal mice during postnatal development and in mutant mice with congenital hearing loss. We further identified a subtype of bushy cells expressing the osteopontin-encoding gene *Spp1* as the primary CN cell type that exhibited hearing loss-induced alteration of gene expression. Among the highly affected genes in bushy cells, deletion of the auditory input-regulated gene *Spp1* affected CN processing of auditory signals in mice. These results provide the most comprehensive cellular and molecular database to date for understanding auditory processing within the CN and identifying potential therapeutic targets for hearing restoration at the CN level.

## Introduction

The auditory system is responsible for processing sounds from the environment by decoding sound frequency, amplitude, and temporal features. It achieves remarkable precision in sound source localization and acoustic object discrimination. Inner hair cells (IHCs) perform the initial sensory transduction of sound stimuli and transmit information to the cochlear nucleus (CN) via auditory nerve fibers.^1^ The CN comprises diverse neuronal types that play a variety of roles in the processing of auditory signals. Some CN neurons are also involved in multisensory integration.^2–4^ The CN serves as the primary central relay in the auditory pathway and is the target for auditory brainstem restoration^5^ in patients who obtain little-to-no benefit from cochlear implants. Extensive efforts^6–15^ have been made to investigate the morphological, electrophysiological, and molecular properties of CN neurons. However, a comprehensive understanding of the changes, characteristics, and spatial distribution of CN cell types in response to auditory input underlying auditory processing is still lacking.

Sensory experience is known to modify the structure and function of neural circuits through activity-dependent neuronal plasticity, including cell type specification, axon/dendritic arborization, and the formation of synaptic connections.^11,16–21^ Previous studies^11,21–26^ have demonstrated auditory activity-dependent development of the CN and plasticity of synapses between the auditory nerve and CN neurons. During postnatal development, CN neurons undergo physiological and morphological changes, including the development of calyceal synapses on bushy cells and conventional bouton synapses on stellate cells.^27–29^ Cochlear removal during the first postnatal week reduces the size and number of CN neurons in mice,^30–34^ and cochlear implantation yields better hearing and speech outcomes in hearing-impaired children younger than 12 months than in older children,^35,36^ indicating that auditory activity plays a significant role in CN development. However, which CN cell types and how the global expression of genes are affected by auditory input remain unknown.

In this study, we identified CN subregions based on global gene expression as well as transcriptome-defined cell types and their spatial distribution within the CN using single-nucleus RNA sequencing (snRNA-seq) and spatially enhanced resolution omics sequencing (Stereo-seq).^37^ This atlas enabled us to identify the *Spp1*-expressing subtype of bushy cells as the critical CN cell type affected by congenital sensorineural hearing loss due to IHC malfunction. *Spp1* expression was correlated with hearing onset in normal mice and was downregulated in hearing loss mice, and genetic deletion of *Spp1* affected CN processing of auditory signals in mice. Thus, our study provides a valuable resource of the molecular atlas of CN, laying the foundation for understanding auditory processing physiology and pathophysiology within the mouse CN and contributing to the design of effective neuro-prostheses for hearing restoration.

## Results

### Spatial transcriptomic mapping of CN subregions

**Fig. 1:**
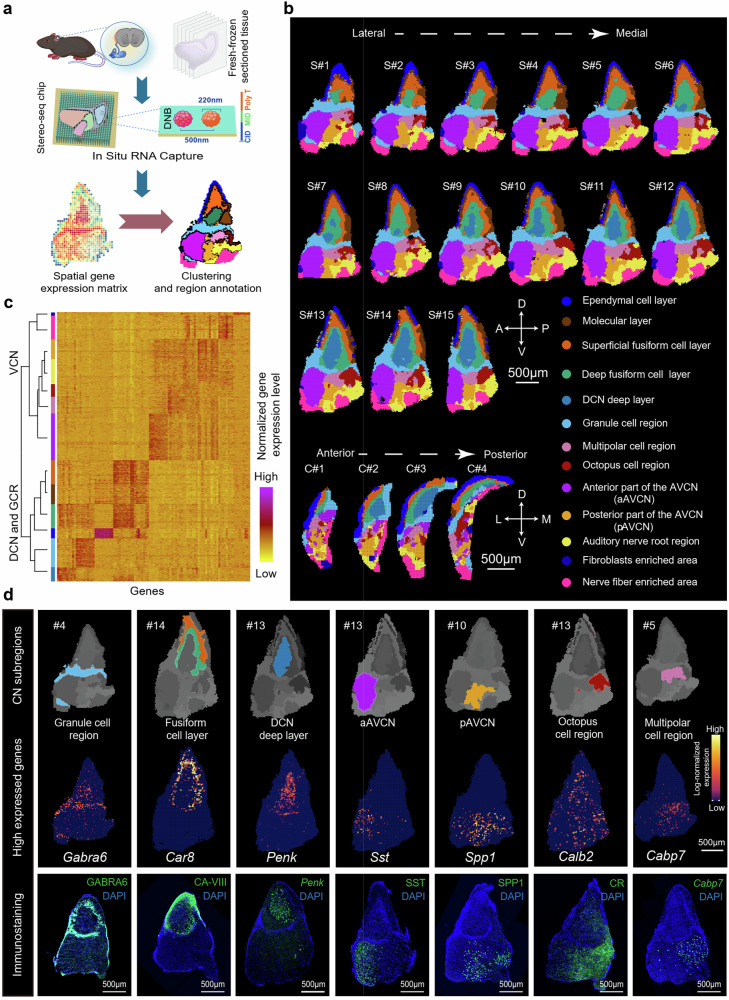
Clustering and annotation of molecularly defined CN subregions. **a** Schematic of Stereo-seq and spatial clustering for the CN. **b** Spatial map of Stereo-seq-defined CN subregions for 15 consecutive sagittal sections (CN #1; S #1–#15) from lateral to medial and 4 coronal sections (CN #6; C #1–#4) from anterior to posterior. Regions are colored on the basis of their spatial transcriptome patterns (A anterior, P posterior, V ventral, D dorsal, L lateral, and M medial; #: numbered sagittal and coronal CN sections). **c** Heatmap showing the normalized expression of marker genes for the indicated CN subregions. **d** Specific colored and labeled subregions of the CN, shown against the background of the other regions in gray (top). Spatial expression of the region-specific marker genes *Gabra6*, *Car8*, *Penk*, *Sst*, *Spp1*, *Calb2*, and *Cabp7* (middle). Immunostaining (GABRA6, CA-VIII, SST, SPP1, and calretinin (CR)) and smFISH (*Penk*, *Cabp7*) images of the identified spatial markers of the CN (bottom).

Previous studies have shown the existence of subregions within the CN.^6,38^ We performed Stereo-seq analysis of the CN from postnatal day 45 (P45) wild-type (WT) mice. We obtained a total of 35 sagittal and coronal sections, including 15 consecutive sagittal sections (from one mouse, CN #1), 16 sagittal sections (at 60-μm intervals from four mice with different starting coordinates in the CN, CN #2–#5), and 4 coronal sections (at 120-μm intervals from one mouse, CN #6). The sections were laid onto Stereo-seq chips, where DNA nanoballs (DNBs; size, 220 nm) were docked onto the chip surface in a grid-patterned array (DNB center-to-center distance, 500 nm). These DNBs captured RNA transcripts, and the sequencing data were processed and integrated to generate a 2D spatial transcriptome map of each section (Fig. 1a). Unsupervised spatially constrained clustering (SCC) was used to group the samples into spatial clusters based on distinct expression profiles and spatial locations of DNBs (see “Materials and Methods”^39^). At a resolution of BIN50 (~25 μm × 25 μm, comprising 50 × 50 DNBs covering multiple adjacent cells), we observed clear CN subregions with distinct gene expression profiles (see Supplementary information, Fig. S1a for different BIN sizes). Typically, the average numbers of unique molecular identifiers (UMIs) and genes at BIN50 resolution were 5823 and 2031, respectively (Supplementary information, Fig. S1b). In total, we identified 13 CN subregions based on spatial transcriptomic patterns (Fig. 1b, c; Supplementary information, Fig. S1c). These transcriptome-defined subregions were highly consistent between adjacent sections and biological replicates (Fig. 1b; Supplementary information, Fig. S1d).

**Fig. 2:**
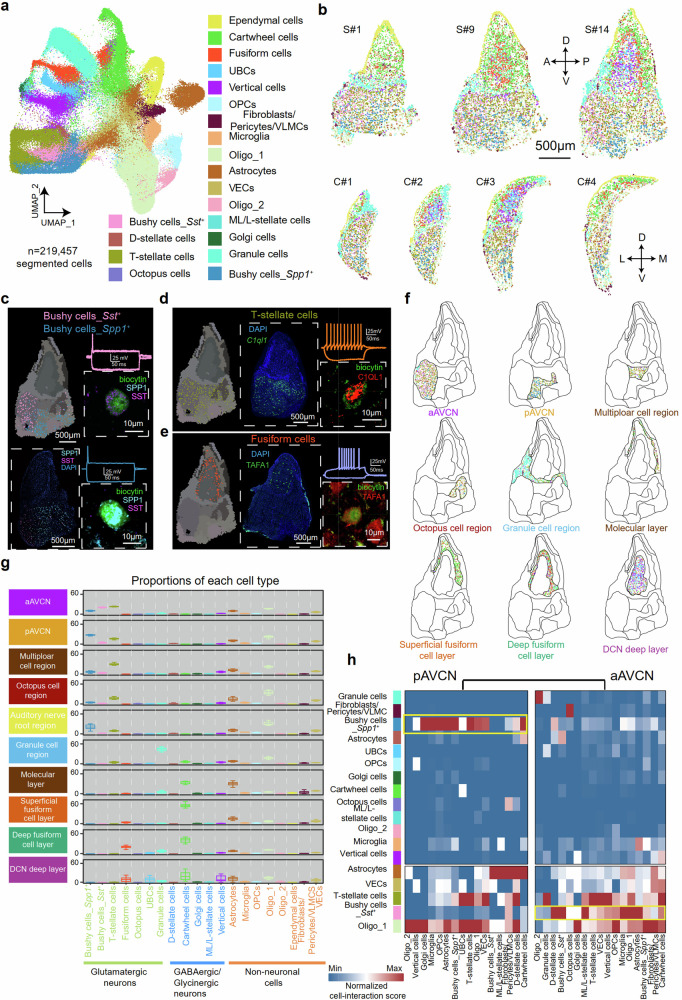
Stereo-seq-defined CN cell types and spatially resolved cell atlas of the mouse CN. **a**, **b** Uniform manifold approximation and projection (UMAP) visualization of segmented cells from all groups of mouse CN sections colored according to their cell type annotations (**a**). Spatial visualization of Stereo-seq-defined cell types in sagittal and coronal sections colored according to their annotations (**b**). **c** Spatial distribution of *Spp1*^*+*^ and *Sst*^*+*^ bushy cells (top left), co-immunostaining of SPP1 and SST (bottom left), patch-clamp recordings (top right), and co-immunostaining of cell type-specific marker proteins with biocytin staining of the recorded cell (bottom right). **d**, **e** Spatial distribution of T-stellate cells (**d**) and fusiform cells (**e**), immunostaining of their marker genes, patch-clamp recordings, and immunostaining of the marker proteins, with biocytin staining of the recorded cell. **f** Overview of the compositional diversity of cell types in each Stereo-seq-defined CN subregion (with the exception of the nerve fiber-enriched area) in a representative sagittal section (contour lines indicate CN subregion boundaries). Cells are colored according to their cell-type identities (**a**). **g** Composition of different cell types (indicated by the percentage of a specific cell type in each layer or subregion) in each Stereo-seq-defined CN subregion. **h** Mean nearest-cell interaction scores of Stereo-seq-defined cell types in the aAVCN and pAVCN. The rectangle indicates the interaction scores between *Spp1*^+^/*Sst*^+^ bushy cells and other cell types. UBCs unipolar brush cells, OPCs oligodendrocyte precursor cells, Oligo oligodendrocytes, VECs vascular endothelial cells, VLMCs vascular and leptomeningeal cells.

We annotated the transcriptome-defined CN subregions that were consistent with histology-based regions using the same names.^6^ The previously described “granular cell region” (GCR) was marked by the granular cell marker gene *Gabra6* (refs.^40,41^; Fig. 1b, c). The CN can be broadly divided into dorsal (DCN) and ventral (VCN) regions on the basis of its cytoarchitecture,^6^ and these regions are believed to perform different CN functions. A previous subdivision of the CN based on cytoarchitectural features showed that the DCN exhibits layered structures.^3^ Our Stereo-seq data enabled molecular identification of these layers. The outermost layer that covers the CN surface, known as the ependymal layer,^42^ expressed high levels of the ependymal cell marker gene *Foxj1* (Supplementary information, Fig. S1e). The molecular layer, which is enriched with many unmyelinated “parallel” fibers and sparsely distributed inhibitory stellate cells,^43,44^ exhibited low expression of the myelin marker *Mbp* and high expression of the GABAergic cell marker *Gad1* (Supplementary information, Fig. S1f). Transcriptomic patterns enabled us to identify subregions within some of these histology-defined regions. The fusiform cell layer was previously defined as the region containing fusiform cells, but it also includes a group of non-fusiform cells known as cartwheel cells, which are Purkinje cell-like inhibitory neurons^45,46^ that express *Car8*. Using a high level of *Car8* expression to define the fusiform cell layer in our transcriptomic map, we found that the gene *Fam19a1* was highly expressed in a subregion of this layer, implying the existence of a further subdivision of the conventional fusiform layer. As shown later, *Fam19a1* was the primary marker gene for the annotation of fusiform cells (see Fig. 2a for cell typing), and the previously histology-defined fusiform layer could be redefined into the “superficial fusiform cell layer” and the “deep fusiform cell layer”. The latter annotation was based on a higher density of fusiform cells than that in the superficial layer (as shown later in Fig. 2g) and is consistent with the finding from physiological recordings that cartwheel cells are encountered before fusiform cells.^47^ Our spatial transcriptome profiles of glutamatergic and GABAergic/glycinergic neurons also support this layer definition based on cartwheel cells (GABAergic/glycinergic) and fusiform cells (glutamatergic) (Fig. 1d; Supplementary information, Fig. S1g). Our spatial gene expression profiles also supported the molecularly defined DCN deep layer, which is known to have a spatially mixed distribution of excitatory neurons (such as unipolar brush cells)^48^ and inhibitory neurons (such as vertical cells)^49^ (Fig. 1d; Supplementary information, Fig. S1h).

Our global spatial transcriptome enabled us to subdivide the VCN into two primary regions based on molecular profiles: the anteroventral CN (AVCN) and the posteroventral CN (PVCN). The AVCN could be further subdivided into anterior (aAVCN) and posterior (pAVCN) regions, which showed high expression of the somatostatin gene *Sst* and the osteopontin (OPN) gene *Spp1*, respectively (Fig. 1b, d). Within the PVCN, we also identified marker genes such as *Cabp7* and *Calb2* for the previously histology-defined multipolar cell region and octopus cell region, respectively (Fig. 1b), and confirmed their protein expression with immunostaining (Fig. 1d). We also identified a subregion corresponding to the previously described “auditory nerve root” region,^50^ which contains mostly glutamatergic neurons (such as bushy cells). A complete list of identified marker genes for CN subregions is provided in Supplementary information, Table S1. In summary, our Stereo-seq data provide a comprehensive molecular fingerprint for more refined definitions of CN subregions.

### Spatial transcriptome of CN cells at single-cell resolution

We next examined the spatial transcriptome map of the CN at single-cell resolution. We performed cell segmentation of the Stereo-seq data based on single-stranded DNA staining that highlighted the nucleus (Supplementary information, Fig. S2a). The watershed algorithm and Gaussian blur algorithm were then used to identify the outlines of individual cells (Supplementary information, Fig. S2a, b). After excluding poorly captured cells, we obtained 219,457 segmented cells with an average of 1861 UMIs and 817 genes per cell (Supplementary information, Fig. S2c). Using Seurat’s unsupervised clustering analysis, we identified 20 major cell types (Fig. 2a; Supplementary information, Fig. S2d; the complete list of differentially expressed genes (DEGs) is available in Supplementary information, Table S2) on the basis of their spatial locations and the expression of known marker genes.^15,51^ The spatial distributions of these 20 cell types are shown in the composite map in Fig. 2b.

**Fig. 3:**
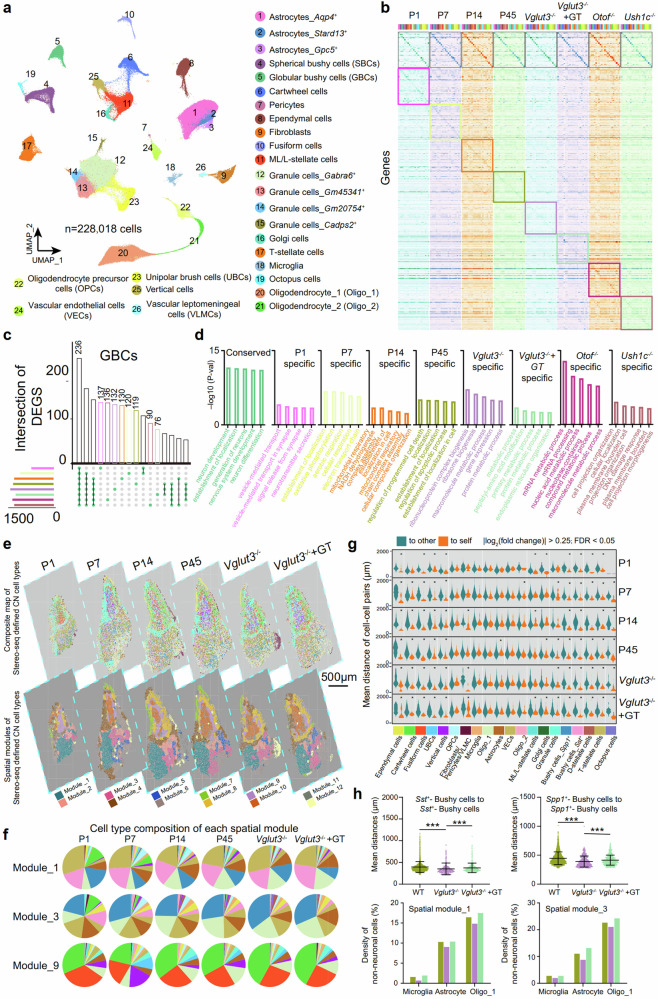
Role of auditory activity-dependent CN cell gene expression and spatial organization. **a** UMAP of CN cells from all groups using snRNA-seq data. Cells are colored according to their cell-type annotations. **b** Heatmap showing the expression of conserved and mouse group-enriched marker genes for each cell type from the snRNA-seq data. **c** UpSet plots showing the numbers of shared and divergent marker genes for GBCs from snRNA-seq data across different mouse groups. **d** GO terms associated with DEGs highly expressed in GBCs from different mouse groups. **e** Spatial maps (top) of Stereo-seq-defined CN cell types from P1, P7, P14, P45 (WT), *Vglut3*^*–/–*^ and *Vglut3*^*–/–*^+GT. Cells are colored according to their cell-type identity (Fig. 2a). Spatial maps of cells, colored according to their spatial module identities (bottom), are shown in representative sagittal sections from different mouse groups. **f** Fraction of cells in three representative spatial modules across different mouse groups. **g** Distribution of mean cell–cell distances within the same cell type (“to self”, red) or between different cell types (“to other”, blue) determined from the Stereo-seq data. False discovery rate (FDR): *P*-value determined by the one-sided Wilcoxon rank-sum test and adjusted to FDR by the Benjamini–Hochberg (BH) procedure. **h** Mean intra-type distances of the two types of bushy cells and the density of glial cells in the two spatial modules in which bushy cells are mainly localized. Statistical analysis was performed using one-way ANOVA followed by a Bonferroni post hoc test. ***P < 0.001.

Based on our Stereo-seq map, bushy cells could be divided into two types, annotated as *Sst*^*+*^ (high *Sst* and low *Spp1* expression) and *Spp1*^*+*^ (high *Spp1* expression and low *Sst* expression), and this was supported by the finding that very few cells were co-immunostained for both SPP1 and SST (Fig. 2c). This division of SST^+^ and SPP1^+^ subtypes largely overlapped with our snRNA-seq-defined subtypes of bushy cells (Supplementary information, Figs. S4g, S6c) — the spherical bushy cells (SBCs) and globular bushy cells (GBCs), as defined by their respective marker genes^15^ *Atoh7* and *Hhip* (Fig. 3a). Co-immunostaining experiments for SPP1 and HHIP, as well as for SST and ATOH7 (Supplementary information, Fig. S2e), confirmed the high-level co-localization of these corresponding proteins. Furthermore, both SPP1^+^ and SST^+^ neurons showed electrophysiological properties characteristic of bushy cells (Fig. 2c). We found that *Sst* was highly expressed in the *Sst*^+^ subtype of bushy cells (known to be excitatory; Supplementary information, Fig. S2f), consistent with previous findings,^15^ despite *Sst* being a well-known marker gene for GABAergic neurons in the brain.^52^ We also identified candidate marker genes for T-stellate cells (*C1ql1*) and fusiform cells (*Fam19a1*), which were confirmed on the basis of cells that could express the corresponding marker proteins and exhibit electrophysiological properties^13^ known to be distinct for these cell types (Fig. 2d, e).

Consecutive and replicated sagittal sections across the CN for Stereo-seq analysis enabled us to obtain a global view of CN cell distribution along the anterior-posterior and medial-lateral CN axes (Supplementary information, Fig. S3a). The spatial maps of individual CN cell types (Supplementary information, Fig. S3b, c) indicated that most neuronal types were distributed with distinct regional preferences, whereas non-neuronal cell types were largely randomly distributed across the CN. Mapping regional cell-type compositions may help determine potential cell–cell interactions underlying the functions of distinct CN cell types. By quantifying the percentages of various cell types among all cells within a local region, we found distinct compositions of glutamatergic and GABAergic/glycinergic neuron types in each DCN layer and VCN subregion (Fig. 2f, g). For example, the superficial fusiform cell layer comprised predominantly cartwheel cells, as well as fusiform cells and astrocytes, whereas the auditory nerve root region comprised *Spp1*^+^ bushy cells, T-stellate cells, and oligodendrocytes, consistent with its proximity to the nerve root.^50^

In addition to revealing the cell-type compositions of various CN subregions, the high-resolution spatial atlas enabled us to infer potential cell–cell interactions. As expected, the interaction matrix for the aAVCN region showed that the closest cell type to *Sst*^*+*^ bushy cells was *Sst*^*+*^ bushy cells themselves (Fig. 2h), consistent with their localized distribution in the aAVCN. Similarly, highly localized *Spp1*^*+*^ bushy cells in the pAVCN region were also revealed by the interaction matrix, indicating a highly clustered distribution of *Spp1*^*+*^ bushy cells in this region (Fig. 2h). More interestingly, microglia displayed high proximity to *Spp1*^*+*^ bushy cells in the pAVCN region but not to *Sst*^*+*^ bushy cells in the aAVCN region (Fig. 2h), suggesting region-specific neuron–non-neuron interactions.

### Role of auditory activity in determining CN gene expression and cell types

To understand the molecular and cellular changes in the CN in response to auditory inputs, we performed snRNA-seq on CN samples from various groups of mice, aiming to reveal the role of auditory activity in gene expression and cell-type determination during development and hearing impairment (Supplementary information, Fig. S4a). To investigate the developmental role of auditory activity, we examined P1 mice when synaptic contacts are established between IHCs and spiral ganglion neurons (SGNs),^53^ P7 mice during the synapse refinement period,^54^ P14 mice shortly after hearing onset,^55^ and P45 mice after auditory maturation (“WT” mice).^55^ We also examined three groups of mice with sensorineural hearing loss due to deletion of specific genes (*Vglut3*^*–/–*^, *Ush1c*^*–/–*^, and *Otof*^*–/–*^ mice)^56–58^ and one group of hearing loss *Vglut3*^*–/–*^ mice after gene therapy with *Vglut3* overexpression (*Vglut3*^*–/–*^*+*GT mice).^56^ The effects of gene deletion and overexpression in these mice were confirmed by auditory brainstem response (ABR) tests (Supplementary information, Fig. S4b).

We performed unsupervised clustering analysis of the snRNA-seq data from a total of 228,018 cells pooled from all groups of mice (Fig. 3a; Supplementary information, Fig. S4c). The cell transcriptome data passed quality control and doublet exclusion, yielding a mean of 4152 UMIs and 1698 genes per cell (Supplementary information, Fig. S4d). The cell types were then annotated using previously identified cell-type marker genes^15,51^ and the marker genes identified in our Stereo-seq analysis (Fig. 2). A total of 26 cell types were identified (Fig. 3a; the complete list of DEGs is available in Supplementary information, Table S3). The annotations were further supported by mapping the snRNA-seq data onto the Stereo-seq data at a resolution of BIN20 (10 μm × 10 μm; Supplementary information, Fig. S4e) using the robust cell-type decomposition (RCTD) procedure.^59^ These transcriptomic data also revealed previously unknown subtypes of CN cells that may play distinct functional roles. For example, we identified three subtypes of astrocytes that showed distinct spatial distributions within the CN (Supplementary information, Fig. S4f). Four different transcriptome-based granular cell subtypes were also identified within the GCR (Supplementary information, Fig. S4f), suggesting that they may have region-specific functions. Furthermore, the marker genes (*Spp1* and *Sst*) of Stereo-seq-defined bushy cells were also expressed in snRNA-seq-defined bushy cells. Specifically, GBCs highly expressed *Spp1*, whereas some SBCs highly expressed *Sst* and others highly expressed *Spp1*, as shown in the snRNA-seq clustering map (Supplementary information, Fig. S4g).

Similar cell types were identified in each mouse group (Supplementary information, Fig. S5a). By performing unsupervised clustering analysis of cells from each mouse group, we found that more than ~95% of cells could be mapped to cell types with the same annotations as those identified in the pooled dataset from all groups (Supplementary information, Fig. S5b, c). This suggests that the cell-type compositions of the CN did not vary significantly among mice at different developmental stages and hearing conditions. We found that the percentages of various cell types were similar between WT and mutant mice (Supplementary information, Fig. S5d), but there were clear changes during CN development (Supplementary information, Fig. S5a). For example, the percentage of Oligo (oligodendrocyte)_1 cells gradually increased, consistent with myelin formation during CN development.^60^

To further determine the characteristic expression patterns of various cell types in each mouse group, we screened for DEGs in each cell type. Notably, a large number of marker genes in each cell type were shared among different mouse groups, indicating that cell-type identity did not change across different ages and genotypes (Fig. 3b). However, a substantial number of DEGs in each cell type were highly expressed in only one mouse group, suggesting differences in gene expression related to CN development and hearing loss. For example, within GBCs, we detected a total of 236 common markers across all groups, which may represent conserved identity genes for this cell type (Fig. 3c). We also identified 940 group-specific DEGs that were highly expressed exclusively in one mouse group (Fig. 3c). We then performed Gene Ontology (GO) analysis of these specific DEGs (Fig. 3d). We found that genes highly expressed in GBCs of the P7 and P14 mouse groups were related to mitochondrial respiratory function, perhaps suggesting stronger auditory input-induced activity in GBCs than in other cell types (Fig. 3d). By contrast, highly expressed DEGs in GBCs from the hearing loss groups were associated with GO terms related to protein or gene metabolic processes, suggesting a possible regulatory role of auditory input in maintaining normal neuronal function. Other cell types, such as fusiform cells, SBCs, and microglia, also exhibited specific cellular properties in response to auditory input (Supplementary information, Fig. S5e–h). For marker genes, we examined their expression in different mouse groups across ages (P1, P7, P14, and P45) and genotypes (*Vglut3*^*–/–*^, *Vglut3*^*–/–*^*+*GT, *Otof*^*–/–*^, *Ush1c*^*–/–*^) (Supplementary information, Fig. S6a, b). Some cell types showed relatively stable expression of marker genes (such as *Fam19a1* and *C1qc*) throughout development (from P1 onward), whereas others gradually exhibited relatively stable expression only after hearing onset (Supplementary information, Fig. S6a, b). In addition, we found high correlation coefficients between the averaged gene expression profiles of each major cell type in the snRNA-seq and Stereo-seq datasets (Supplementary information, Fig. S6c). Together, our study provided a more global atlas of gene expression patterns in the CN with or without auditory input.

### Spatial transcriptomes reveal activity-dependent changes in cellular organization

We next performed spatial transcriptome analysis to examine the effect of auditory activity on the spatial organization of various cell types within the CN (Fig. 2; Supplementary information, Fig. S7). Stereo-seq analyses were performed on mice at four developmental stages (P1, P7, P14, and P45), hearing loss gene-deletion mice (*Vglut3*^*–/–*^ mice), and *Vglut3*^*–/–*^ mice whose hearing was restored by *Vglut3* overexpression (*Vglut3*^*–/–*^*+*GT mice).

The cellular organization of various cell types within the CN was further examined by determining spatial modules containing cells with similar neighborhood cell-type compositions using BANKSY (Fig. 3e).^39^ The spatial modules segmented the CN into areas that largely coincided with the major CN subregions defined by our Stereo-seq data (Fig. 1). The spatial modules of the CN and their cell-type compositions were similar between WT and hearing-impaired mice (Fig. 3f). During postnatal development, the auditory nerve input may influence the cellular organization within spatial modules, which emerged during CN maturation. From P1 onward, we could identify putative CN spatial modules resembling those in the adult CN. In addition, the cell-type compositions of these spatial modules were similar among mice at different developmental stages (Fig. 3f).

Further examination of nearest-neighbor distances among various cell types also revealed potential effects of auditory activity on cellular organization in the CN. We examined the distances between individual cells and their nearest neighbors of the same or different cell type across the entire CN (Fig. 3g). Neuronal cells, such as fusiform cells and bushy cells, already showed a tendency for self-affinity during postnatal development and in mutant mice with congenital hearing loss (Fig. 3g). This suggests that the self-affinity characteristic of neuronal cells is unaffected by auditory input in the CN. However, by comparing the nearest-neighbor distances between cells of the same type (“intra-type” distances) in mice with hearing loss and WT mice, we found that the intra-type distances for bushy cells were reduced in *Vglut3*^*–/–*^ mice. Furthermore, WT intra-type distances were partially restored in *Vglut3*^*–/–*^ mice whose hearing was restored by *Vglut3* overexpression (Fig. 3h). The reduction in intra-type distances between these neurons in hearing loss mice is likely attributable to the reduced density of non-neuronal cells (Fig. 3h).

### Hearing loss primarily affected *Spp1*^*+*^ bushy cells

We used individual loss-of-function mutations of *Ush1c*, *Otof*, and *Vglut3* (*Slc17a8*) to generate hearing loss mouse models. *Ush1c* encodes the scaffolding protein harmonin, which contributes to the sensitivity of mechano-transduction channels in response to hair-bundle displacements.^58^ *Otof* encodes otoferlin, the key protein that mediates vesicle release at hair-cell ribbon synapses.^57^ *Vglut3* encodes the vesicular glutamate transporter VGluT3.^61^

We examined the effect of hearing loss on genome-wide expression profiles of various CN cell types by identifying DEGs between normal and hearing loss animals using snRNA-seq data (Supplementary information, Fig. S8a). We found that the number of auditory input-dependent DEGs was generally greater in neuronal cell types than in non-neuronal cells (Supplementary information, Fig. S8a). We found that 187 DEGs were common among these group pairs and could be classified into two or four modules (Supplementary information, Fig. S8b, c). Further analysis using the Search Tool for the Retrieval of Interacting Genes (STRING; https://string-db.org) showed significant interactions among these modules (Supplementary information, Fig. S8d, e), some of which may be related to specific signaling pathways, as suggested by GO analysis. Thus, specific gene regulatory networks may function in an auditory input-dependent manner. Furthermore, analysis using the irGSEA method^62^ showed that, among all cell types, bushy cells (GBCs and SBCs) had the highest enrichment scores for these up- and downregulated genes (Supplementary information, Fig. S8f), indicating that changes in gene expression patterns were most pronounced in bushy cells.

**Fig. 4:**
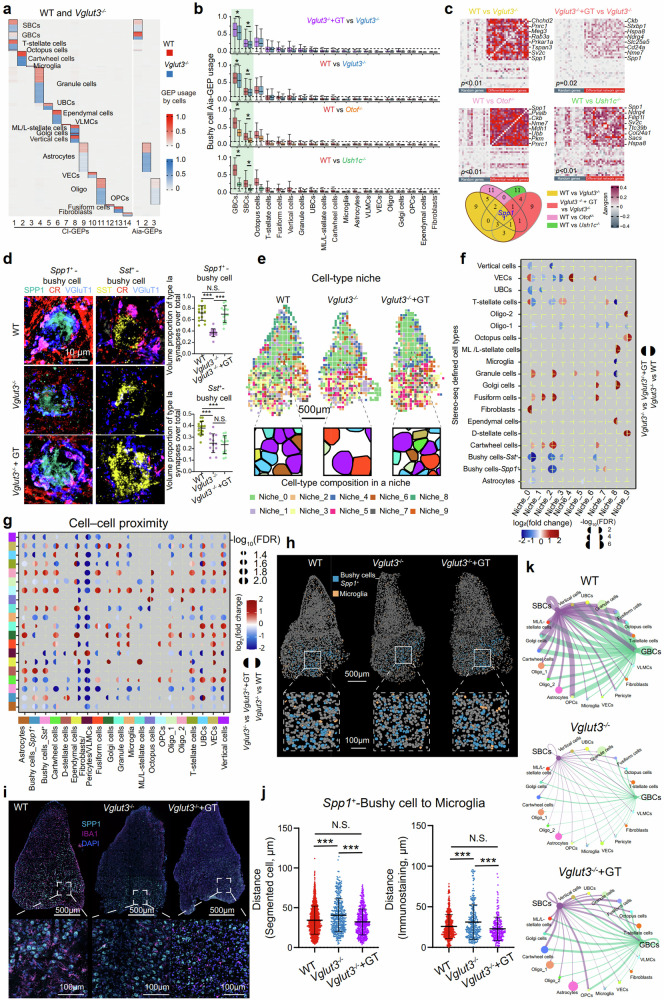
*Spp1*^+^ bushy cells were identified as the primary cell type that showed transcriptomic and spatial relationship responses to auditory stimuli. **a** Heatmap showing the fractional usage of all GEPs (columns) in all cells (rows). CI-GEPs are shown on the left and Aia-GEPs on the right. Cells are grouped by their annotations based on snRNA-seq data and fit into columns of fixed width for each CI-GEP. **b** Box-and-whisker plots (central line represents the median, boxes represent the interquartile range, and whiskers represent the 5th and 95th quantiles) showing the percentage usage of Aia-GEPs in different cell types. The dashed line represents 10% usage of activity programs. Statistical analysis was performed using the Wilcoxon rank-sum test. *P < 0.05. **c** Heatmaps showing the differential network genes of bushy cells in different comparative studies using snRNA-seq data. The Venn diagram shows *Spp1* as the only overlapping gene. **d** Immunostaining of the afferent synapse and the type Ia endbulb of the Held synapse of the two types of bushy cells in WT, *Vglut3*^*–/–*^ and *Vglut3*^*–/–*^+GT mice, and quantification of the changes in the volume proportion of the type Ia endbulb of the Held synapse in the two types of bushy cells. Statistical analysis was performed using one-way ANOVA followed by a Bonferroni post hoc test. N.S., *P* > 0.05; ***P < 0.001. **e** Spatial mapping of cell-type niches in different mouse groups (top), and the cell-type composition in a niche (50 μm × 50 μm). **f** Differences in cell-type composition in a niche are shown in a semi-dot plot. The left and right halves of each semi-dot indicate the significant cell-type composition in a niche of *Vglut3*^*–/–*^+GT vs *Vglut3*^*–/–*^ and WT vs *Vglut3*^*–/–*^ mice, respectively. The color represents the log_2_(fold change) of a cell type, and the dot size indicates the significance. Statistical analysis was performed using the Wilcoxon rank-sum test. **g** Enrichment of cell–cell proximity of different cell types is shown in a semi-dot plot using Stereo-seq data. The left and right halves of each semi-dot indicate the significant cell–cell proximity of *Vglut3*^*–/–*^+GT vs *Vglut3*^*–/–*^ and WT vs *Vglut3*^*–/–*^ mice, respectively. The color represents the log_2_(fold change) of the colocalization frequency of the two cell types, and the dot size indicates the significance of the colocalization. Statistical analysis was performed using the Wilcoxon rank-sum test. **h**, **i** Representative sections showing the locations of *Spp1*^+^ bushy cells and microglia from Stereo-seq data (**h**) and immunostaining (**i**) in WT and *Vglut3*^*–/–*^ mice. **j** Quantification of the nearest-neighbor distance (Stereo-seq and immunostaining data, right) between *Spp1*^+^ bushy cells and microglia. Statistical analysis was performed using the Wilcoxon rank-sum test. N.S., *P* > 0.05; ***P < 0.001. **k** CellChat networks of snRNA-seq-defined GBCs and SBCs to other cell types, with the bandwidth representing the communication strength. OPCs oligodendrocyte precursor cells, Oligo oligodendrocytes, VECs vascular endothelial cells, VLMCs vascular and leptomeningeal cells, UBCs unipolar brush cells.

To further characterize transcriptomic changes in mutant mice, we analyzed gene expression programs (GEPs)^63^ that may represent different functional states of CN cells related to auditory stimulation and are distinct from GEPs underlying cell-type identity (CI-GEPs). Using snRNA-seq analysis of CN tissues obtained from WT and *Vglut3*^*–/–*^ mice, we identified 17 distinct GEPs, 14 of which corresponded to CI-GEPs and 3 to non-identity GEPs (Fig. 4a; Supplementary information, Table S4). Notably, all CN neuronal types showed high usage of CI-GEPs, whereas only bushy cells used one of the three non-identity programs. By comparing the usage of these novel GEPs in CN cells between WT and *Vglut3*^*–/–*^ mice, we defined auditory input-associated GEPs (Aia-GEPs) on the basis of their selective occurrence in the same CN cell type in both groups of mice. Notably, the usage of non-identity GEP-1 in bushy cells was significantly higher in WT mice than in all three hearing loss mouse models (*Vglut3*^*–/–*^, *Otof* ^*–/–*^, and *Ush1c*^*–/–*^) (Fig. 4b; Supplementary information, Fig. S9a–c). Furthermore, non-identity GEP-1 usage was partially recovered in *Vglut3*^*–/–*^*+*GT mice (Fig. 4b; Supplementary information, Fig. S9d, e). Thus, non-identity GEP-1 in bushy cells is related to auditory input. By contrast, CN astrocytes and oligodendrocytes showed much higher usage of non-identity GEP-Astro (astrocyte) and GEP-Oligo in *Vglut3*^*–/–*^ mice than in WT mice, respectively. However, such higher usage was not observed in the other hearing loss mice (Supplementary information, Fig. S9f). Therefore, bushy cells represent the primary cell type that showed the most prominent transcriptomic response to auditory nerve input. This finding was further supported by the observation that transcriptomic differences (“transcriptional shifts”^64^) in bushy cells were the largest among all CN cell types, and these shifts were significant between WT mice and all three types of hearing loss mice, with the exception of *Vglut3*^*–/–*^+GT mice (Supplementary information, Fig. S9g–l).

Intrigued by the transcriptional changes observed in bushy cells, we performed a more in-depth molecular characterization of these cells in response to hearing loss. We first compared cell-specific gene networks to identify differentially regulated genes that might play crucial roles in network regulation between WT and hearing loss mice (Fig. 4c). This was accomplished by constructing a network degree matrix for bushy cell genes, with nodes and edges representing gene–gene dependencies, allowing for nonparametric estimation of co-expressed genes between WT and hearing loss mice. Among all differentially expressed network genes, *Spp1* was the only gene that differed between WT mice and all three groups of hearing loss mice, except for *Vglut3*^*–/–*^+GT mice (Fig. 4c; Supplementary information, Fig. S9m), suggesting that *Spp1* plays an essential role in the auditory functions of bushy cells. In addition, *Spp1* expression was downregulated in hearing loss mice (*Vglut3*^*–/–*^, *Ush1c*^*–/–*^, and *Otof*^*–/–*^ mice) but upregulated after hearing restoration (*Vglut3*^*–/–*^+GT) (Supplementary information, Fig. S10a, b). We also observed fewer SPP1^+^ cells in hearing loss mice than in WT mice, which was partially restored to WT levels after hearing restoration, as demonstrated by immunostaining (Supplementary information, Fig. S10c, d). This is consistent with the notion that *Spp1* expression in bushy cells is enhanced in response to acoustic stimuli. Since *Spp1* was the primary marker gene for Stereo-seq-defined bushy cells (Fig. 2), we concluded that *Spp1*^*+*^ bushy cells were the primary cell type showing gene expression changes in all mutant mice.

To further explore how hearing loss affects *Spp1*^*+*^ bushy cells, we measured and analyzed the volume proportion of Ia and non-Ia auditory endbulbs of Held synapses. The proportion of Ia auditory endbulbs, which provide more robust acoustic inputs from the auditory nerve to *Spp1*^*+*^ bushy cells,^65^ was significantly lower in *Vglut3*^*–/–*^ hearing loss mice than in WT mice, but not in *Vglut3*^*–/–*^ mice whose hearing was restored by reintroduction of *Vglut3* (Fig. 4d). The proportion of non-Ia auditory endbulbs showed a corresponding increase in hearing loss mice but returned to WT levels in hearing-restored *Vglut3*^*–/–*^+GT mice (Supplementary information, Fig. S10e). Such hearing loss-dependent effects were not observed for Ia or non-Ia auditory endbulbs of Held synapses on *Sst*^*+*^ bushy cells (Fig. 4d; Supplementary information, Fig. S10e). In addition, the average soma size of *Spp1*^*+*^ bushy cells was smaller in hearing loss mice, whereas this change was not observed for *Sst*^*+*^ bushy cells (Supplementary information, Fig. S10f–i).

Our Stereo-seq data indicated that the regional composition of cell types was not changed in hearing loss mice (Fig. 3e; Supplementary information, Fig. S7). We quantified the spatial expression patterns of *Spp1* and *Sst* in bushy cells and found that *Spp1* expression was downregulated in *Vglut3*^*–/–*^ mice compared with WT mice but was partially recovered in *Vglut3*^*–/–*^+GT mice (Supplementary information, Fig. S10j, k). This supports the notion that auditory input affects *Spp1* expression without altering the composition of bushy cells in the CN (Supplementary information, Fig. S5d).

To explore changes in the spatial organization of CN cells in mutant mice, we performed unsupervised clustering based on the cell-type compositions at a resolution of BIN100 (~50 μm × 50 μm) in our Stereo-seq data, which we defined as major cell-type niches (Fig. 4e; Supplementary information, Fig. S11a, b). Each niche was dominated by different cell types (Supplementary information, Fig. S11b). By comparing the numbers of cell types in each niche between normal and hearing-impaired mice, we observed that the organization of CN cellular neighborhoods was altered. For example, the percentage of *Spp1*^+^ bushy cells in Niche 2 was decreased in *Vglut3*^*–/–*^ mice but increased after hearing restoration (*Vglut3*^*–/–*^+GT mice) (Fig. 4f; Supplementary information, Fig. S11c). Furthermore, nearest-neighbor distance analysis showed that the absence of auditory input in *Vglut3*^*–/–*^ mice resulted in changes in the proximity of specific pairs of cell types (Fig. 4g). Among all neuronal types examined, *Spp1*^*+*^ bushy cells exhibited the largest number of cell-type pairs that showed activity-dependent proximity changes (Fig. 4g). For example, we found that the average distance between *Spp1*^*+*^ bushy cells and non-neuronal cells, particularly microglia, was altered based on our single-cell spatial transcriptome map and immunostaining of CN sections (Fig. 4h–j; Supplementary information, Fig. S11d). We also performed cell–cell communication analysis^66^ using snRNA-seq data and observed that the “communication strength”^66^ between snRNA-seq-defined bushy cells and microglia (or astrocytes) was lower in *Vglut3*^*–/–*^ mice than in WT mice and slightly increased in *Vglut3*^*–/–*^+GT mice (Fig. 4k; Supplementary information, Fig. S11e). Taken together, these findings demonstrate that *Spp1*^+^ bushy cells were the primary cell type that exhibited changes in gene expression and spatial relationships with other cell types in all mutant mice.

To elucidate the regulatory mechanisms controlling *Spp1*, we performed single-nucleus ATAC-seq (snATAC-seq) on the CN and annotated the major cell types (Supplementary information, Fig. S12a). We identified three differential chromatin accessibility peaks (Peak1–Peak3) near the *Spp1* locus in bushy cells compared with non-bushy cells (Supplementary information, Fig. S12b), and we predicted 22 transcription factor (TF) binding motifs (Supplementary information, Fig. S12c) within these regions. To further characterize the *Spp1*-associated regulon, we applied SCENIC^67^ to snRNA-seq data from bushy cells. The results revealed two TFs predicted by snATAC-seq (Supplementary information, Fig. S12c), *Nfe2l1* and *Zbtb7a*, that exhibited high regulon specificity scores and were predicted by SCENIC to regulate *Spp1* expression. Furthermore, the expression patterns of *Nfe2l1* and *Zbtb7a* paralleled that of *Spp1*, showing upregulation during development and downregulation in the absence of auditory input. These results further confirm that *Spp1* is an activity-dependent gene.

### Gene expression changes and abnormal auditory processing in *Spp1*^*–/–*^ mice

**Fig. 5:**
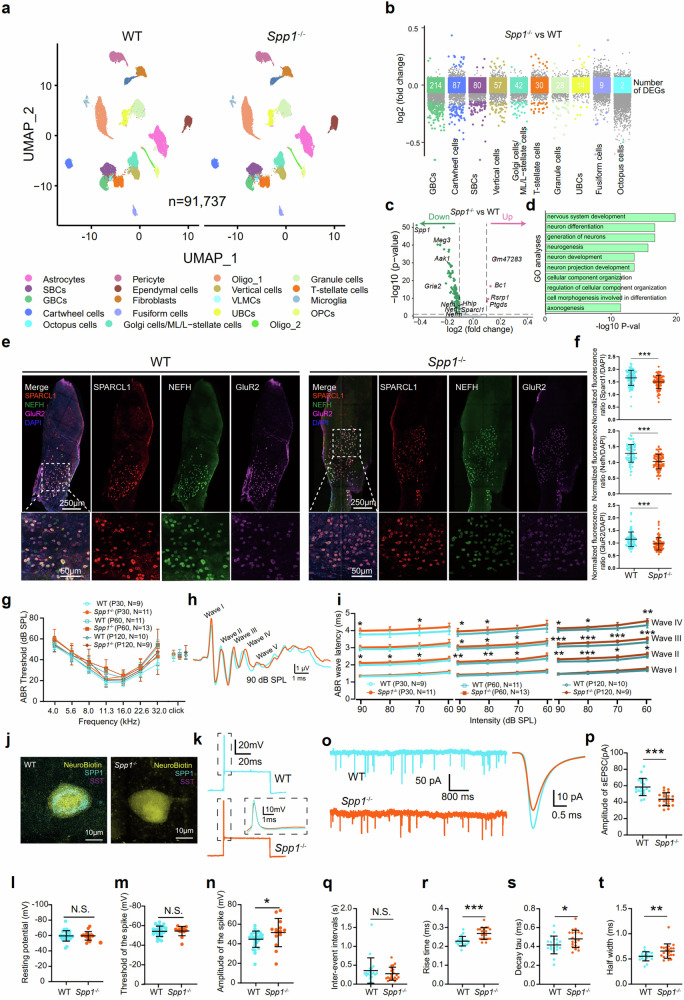
*Spp1* is required to maintain the normal function of the *Spp1*^*+*^ bushy cells. **a** UMAP of the snRNA-seq cell-type taxonomy of the CN from adult WT and *Spp1*^*–/–*^ mice. **b** Strip chart showing DEGs across neuronal types. Genes with colored dots are significantly (*P* < 0.05 and log_2_(fold change) > 0.1) upregulated or downregulated. Genes in gray are not significantly changed. **c**, **d** Volcano plot showing the DEGs of bushy cells between WT and *Spp1*^*–/–*^ mice (**c**) and their GO enrichment analysis (**d**). **e**, **f** Immunostaining showing that the expression of SPARCL1, NEFH, and GluR2 is reduced in *Spp1*^*–/–*^ mice. Statistical analysis was performed using a two-tailed unpaired Student’s *t*-test. ***P < 0.001. **g** *Spp1*^*–/–*^ mice exhibited no significant increase in the ABR threshold (*P* > 0.05, two-way ANOVA followed by a Bonferroni post hoc test). **h** Click-evoked ABR example waves were recorded at 90 dB sound pressure level (SPL) for WT and *Spp1*^*–/–*^ mice, and waves corresponding to waves I, II, III, IV, and V are marked. **i** Latencies of ABR waves II, III, and IV were significantly increased in *Spp1*^*–/–*^ mice at 1 month, 2 months, and 4 months of age. Statistical analysis was performed using two-way ANOVA followed by a Bonferroni post hoc test. *P < 0.05, **P < 0.01, and ***P < 0.001. **j** Images of a recorded neuron that was filled with Neurobiotin (yellow) and immunostained with SPP1 (light blue) and SST (magenta). In WT mice, bushy cells that were exclusively labeled with SPP1 (not expressing SST) were identified as *Spp1*^+^ bushy cells. In *Spp1*-knockout mice, where SPP1 expression is absent, bushy cells (based on their electrophysiological properties) that showed negative SST immunostaining were classified as *Spp1*^+^ bushy cells. **k**–**n** Diagrams showing patch-clamp recordings of the spiking properties of *Spp1*^*+*^ bushy cells (**k**). Comparisons of resting membrane potential (**l**), threshold (**m**), and amplitude (**n**) of the spikes between WT and *Spp1*^*–/–*^ mice are shown. Statistical analysis was performed using a two-tailed unpaired Student’s *t*-test (**i**, **m**) or Mann–Whitney *U* test (**n**). N.S., *P* > 0.05, *P < 0.05. **o** Examples of sEPSC events (left) and average sEPSC traces (right) from *Spp1*^*+*^ bushy cells of WT and *Spp1*^*–/–*^ mice. **p**–**t** Summary of average sEPSC amplitude (**p**), sEPSC event frequency (**q**), 10%–90% rise time (**r**), characteristic decay time (**s**), and half-width (**t**) in WT and *Spp1*^*–/–*^ mice. Statistical analysis was performed using a two-tailed unpaired Student’s *t*-test (**p**, **r**, **t**) or Mann–Whitney *U* test (**q**, **s**). N.S., *P* > 0.05; *P < 0.05, **P < 0.01, and ***P < 0.001.

To examine the importance of *Spp1* expression for CN functions and auditory processing, we generated *Spp1*^*–/–*^ mice by deleting exons 4–7 of *Spp1* and performed snRNA-seq analysis to assess changes in transcriptomic profiles. In total, we obtained 91,737 high-quality nuclei (Supplementary information, Fig. S13a–c) from *Spp1*^*–/–*^ and WT mice (age- and sex-matched littermate controls). Clustering analysis of the transcriptomic profiles revealed no detectable differences in cell types between WT and *Spp1*^*–/–*^ mice (Fig. 5a). As expected, SPP1 protein expression was eliminated (Supplementary information, Fig. S13d). Global transcriptional changes for each CN cell type were examined by DEG analysis between *Spp1*^*–/–*^ and WT mice. We found that snRNA-seq-defined bushy cells exhibited the largest number of DEGs (Fig. 5b; Supplementary information, Fig. S13e). GO analysis of these DEGs demonstrated that genes with reduced expression in bushy cells (Fig. 5c) were associated with GO terms such as “nervous system development”, “neuron projection development”, and “generation of neurons” (Fig. 5d). Immunostaining and western blot analyses confirmed the downregulation of proteins encoded by genes^68^ known to interact with *Spp1* (i.e., *Sparcl1*) and genes involved in neuronal morphology (the neurofilament gene *Nefh*) and synapse development (*Gria2*) (Fig. 5e, f; Supplementary information, Fig. S13e, f). Moreover, we also found that soma size was reduced in *Spp1*^*–/–*^ mice (Supplementary information, Fig. S13g, h).

We next examined whether *Spp1* expression is required for normal auditory information processing in the CN using the ABR test. The hearing threshold remained unchanged in *Spp*^*–/–*^ mice for tested sounds across all pure-tone frequencies and mixed-frequency clicks (Fig. 5g), consistent with a previous finding that the absence of SPP1 in the inner ear does not alter the sensory threshold.^69^ ABR waves comprise several components: wave I represents neural activity in auditory nerve fibers, and waves II–IV reflect neural activity along the pathway from the CN to the inferior colliculus (Fig. 5h).^70^ We found significant delays in the latencies of wave II in *Spp1*^*–/–*^ mice compared with WT mice after hearing onset (at 1 month, 2 months, and 4 months of age; Fig. 5i). The latencies of waves III and IV were also increased in *Spp1*^*–/–*^ mice (Fig. 5i), possibly because SPP1 is also expressed in the superior olivary complex and inferior colliculus (Supplementary information, Fig. S13i). By contrast, wave I latency was not altered, consistent with the absence of SPP1 expression in IHCs and SGNs (Supplementary information, Fig. S13j).

In addition to ABR recording, we also examined the electrophysiological properties of *Spp1*^+^ bushy cells from WT and *Spp1*^*–/–*^ mice (Fig. 5j–t). We observed a significantly larger spike amplitude, lower average amplitude of spontaneous excitatory synaptic currents (sEPSCs), longer rise time and decay time, and smaller half-width of sEPSCs in *Spp1*^*–/–*^ mice compared with WT mice. By contrast, the resting membrane potential, spike threshold, and sEPSC frequency were comparable between WT and *Spp1*^*–/–*^ mice. DEG analysis indicated that oligodendrocytes, astrocytes, and microglia were also affected by *Spp1* deletion (Supplementary information, Fig. S13k). Taken together, these results further support the notion that *Spp1* expression plays a significant role in auditory information processing in the CN.

### *Spp1* expression and proximity of *Spp1*^+^ bushy cells during CN maturation

**Fig. 6:**
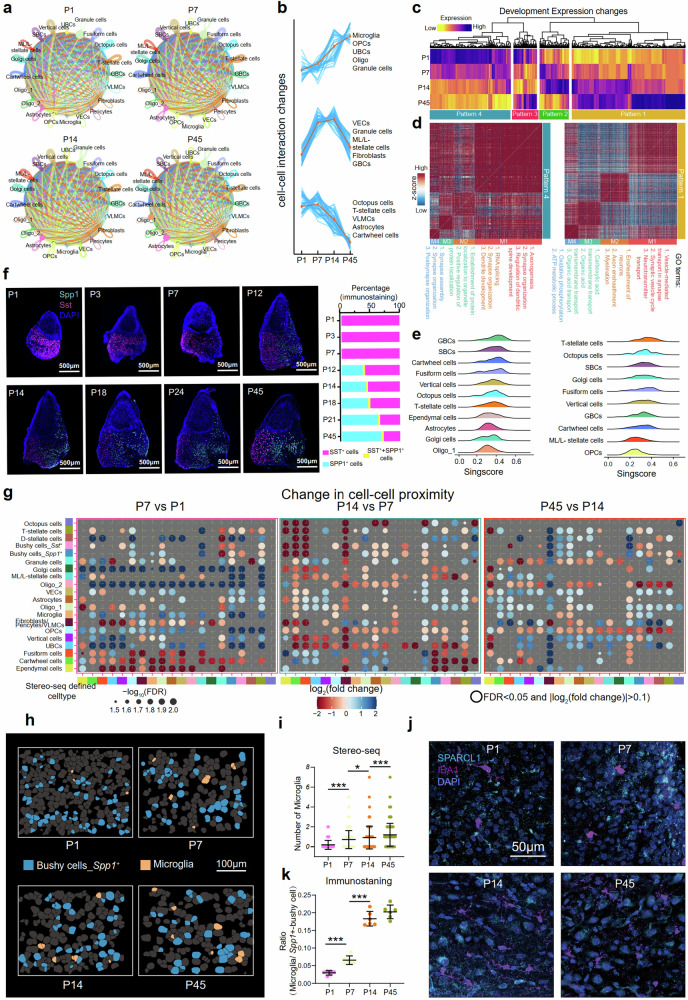
Developmental changes in gene expression profiles and spatial distribution of cell types. **a** CellChat networks between different snRNA-seq-defined CN cell types, with the bandwidth representing the communication strength at different developmental stages. **b** Line plot depicting dynamic changes in cell–cell communication strength by fuzzy cluster analysis for three groups, with cell types (top 5) in each dynamic pattern listed. **c**, **d** Heatmaps showing the gene modules of genes from patterns 1 and 4, respectively. Selected GO terms associated with representative modules are shown at the bottom. **e** Ridge plot showing enriched expression of pattern 1 and 4 gene sets across different snRNA-seq-defined cell types using kernel density curves (top 10 cell types). **f** Expression of SPP1 and SST by immunostaining at eight ages is shown on the left, and the relative proportion of cells expressing the two proteins is shown on the right. **g** Differential enrichment of cell–cell proximity across four developmental stages. The color represents the log_2_(fold change) of the colocalization frequency of the two cell types, and the dot size indicates the significance of the colocalization. Statistical analysis was performed using the Wilcoxon rank-sum test. **h**–**k** Zoomed-in regions showing the proximity of *Spp1*^+^ bushy cells and microglia from the Stereo-seq data (**h**) and immunostaining (**j**) during development. Quantification of the number of microglia in 30 neighboring cells of an *Spp1*^+^ bushy cell (**i**) and the ratio of microglia to *Spp1*^+^ bushy cells in the pAVCN region (**k**). Statistical analysis was performed using one-way ANOVA followed by a Bonferroni post hoc test. *P < 0.05 and ***P < 0.001.

In most rodents, auditory function develops after birth, with a gradual onset of hearing around P12 and maturation around one month of age,^55^ in line with postnatal refinement of the auditory system in response to auditory inputs. We first examined changes in cell–cell interactions during postnatal development by performing CellChat^66^ analysis of the snRNA-seq data. We found that the communication strength among all cell types gradually increased and then decreased (Fig. 6a; Supplementary information, Fig. S14a). We then classified the communication strength into three clusters: gradual increase (cluster 1), biphasic increase followed by decrease (cluster 2), and gradual decrease (cluster 3) across different developmental stages (Fig. 6b). These results suggest that cell–cell communication is likely regulated by hearing-related activity, as the communication strength of microglia with other cells gradually increased, consistent with the finding that auditory input can increase their interactions with bushy cells (Fig. 4h). We then examined changes in gene expression during postnatal development using snRNA-seq data. On the basis of temporal expression changes, we categorized the DEGs into four patterns (Fig. 6c; Supplementary information, Fig. S14b–e and Table S5): gradually upregulated (pattern 1), gradually downregulated (pattern 4), biphasic up–down (pattern 2), and biphasic down–up (pattern 3). Patterns 2 and 3 exhibited sharp transitions around the onset of hearing (P14), suggesting that their regulation may be influenced by auditory experience.

We next examined correlated expression of these genes in the form of matrix modules by hotspot analysis of each pattern, and identified gene modules associated with each of the four DEG patterns (Fig. 6d; Supplementary information, Fig. S14b–e) and their related GO terms. GO terms for pattern 1 modules were associated with synaptic activity, ensheathment of neurons, and ATP metabolic processes (Fig. 6d), whereas pattern 4 modules were associated with GO terms for axonogenesis, development, protein localization, and RNA splicing (Fig. 6d). The processes associated with module 1 and module 4 genes are likely to be up- and downregulated, respectively, during the gradual increase in activity. We also investigated the cell types that were enriched in genes that exhibited up- and downregulation during development (Fig. 6e; Supplementary information, Fig. S14c, e). Bushy cells (GBCs and SBCs) exhibited the highest enrichment scores for pattern 1 genes among all cell types (Fig. 6e), consistent with our finding that bushy cells are the primary cell types affected by auditory nerve input (Fig. 4). On the other hand, genes that were downregulated during development (pattern 4) were mainly enriched in T-stellate cells, octopus cells, and SBCs (Fig. 6e).

Pseudotime analysis revealed a clear separation of GBCs and T-stellate cells at different developmental stages along the predicted pseudotime progression (Supplementary information, Fig. S14f–k). Notably, *Spp1* is a pseudotime marker gene of GBCs that was upregulated in line with the auditory nerve input (Supplementary information, Fig. S14f–h). We next performed immunostaining for SPP1 and SST across various developmental stages and found a significant increase in the number of SPP1^+^ cells around the time of hearing onset (Fig. 6f). Our snRNA-seq data showed that while the number of bushy cells remained constant during development, those expressing *Spp1* gradually increased (Supplementary information, Fig. S5c). Thus, the increased number of SPP1^+^ cells was likely due to increased *Spp1* expression in bushy cells as a result of auditory input.

Finally, our Stereo-seq data also enabled us to map the spatial organization of individual cell types across different developmental stages (Supplementary information, Fig. S7). For each cell type, we measured spatial neighborhood cell–cell interactions and found that the proximity of cell types showed gradual changes during development (Fig. 6g), suggesting developmental modulation of heterologous cell–cell interactions. For example, at P7, most cells were surrounded by OPCs or Oligo_2 cells, a phenomenon not observed at P14 or P45, suggesting active oligodendrocyte development and maturation during earlier stages. In addition, *Spp1*^+^ bushy cells were surrounded by an increasing number of non-neuronal cells, such as microglia, throughout development (Fig. 6g–k; Supplementary information, Fig. S14l), in line with the increased “communication strength” between microglia and snRNA-seq-defined bushy cells (Supplementary information, Fig. S14m). Moreover, the proximity of *Spp1*^+^ bushy cells to other neuronal types, such as T-stellate cells, decreased during development (Fig. 6g). This mapping of the spatial organization of specific CN cell types during development provides a basis for further studies on the role of cell–cell interactions and auditory activity in the development and modulation of CN organization.

## Discussion

In this study, we generated a comprehensive transcriptome-based cell atlas of the mouse CN using snRNA-seq and Stereo-seq approaches. We characterized the gene expression profiles of various CN subregions, identified transcriptome-defined cell types, and mapped their spatial distributions within the CN. Through comprehensive transcriptome analysis, we found that *Spp1*^+^ bushy cells were the primary cell type showing the most prominent transcriptomic response to auditory nerve input. Furthermore, we identified an auditory activity-dependent gene, *Spp1*, that is necessary for maintaining normal auditory processing functions. Together, our study provides a basis for a molecular and cellular understanding of the development of the CN and hearing-dependent abnormalities, as well as for the development of therapeutic treatments for hearing disorders.

### Molecular definition of CN subregions and identification of cell types

Stereo-seq was used to generate a spatial transcriptomic map of the CN and to characterize molecular fingerprints for previously defined CN subregions such as the GCR, AVCN, and DCN. These data also provided evidence for superficial vs deep subdivisions of the fusiform cell layer. The subregion- and layer-specific gene expression profiles described here complement previous anatomical and histological studies of the CN and provide essential information for future mapping of structural and functional connectivity at single-cell resolution.

We constructed a comprehensive CN cell atlas using our Stereo-seq data and snRNA-seq data. For example, owing to the accessibility of both nuclear and cytoplasmic transcripts,^37,71^ the Stereo-seq data helped classify the snRNA-seq-defined bushy cells into two subtypes based on the expression of *Spp1* and *Sst*. We also identified a candidate marker gene for fusiform cells, which were not detected in a previous study.^15^ Although the number of cells in the snRNA-seq and Stereo-seq data was sufficient to cover all major cell types, we failed to identify previously reported giant cells. This could be attributed to the low number of these cells or their transcriptomic similarity to other cell types. Previous identification of CN neuronal types was based on biophysical and morphological properties.^6,13^ Our work represents an important step in matching these properties with gene expression profiles of the various cell types.

### Auditory input-dependent changes in spatial organization and transcriptomic profiles

Our Stereo-seq data revealed that various CN subregions exhibit distinct transcriptomic profiles, supporting the notion of specific functions associated with each CN subregion. Our analysis of spatial relationships among various cell types also showed hearing-dependent changes in the proximity between various cell types. Interestingly, *Spp1*^*+*^ bushy cells showed closer proximity to microglia in WT mice than in hearing loss mice, suggesting hearing-dependent recruitment of microglia for *Spp1*^*+*^ bushy cell activity. This is consistent with the finding that neuronal activity can recruit microglia to its vicinity.^72,73^ We found that the spatial relationships among various cell types changed markedly during development. This may result from postnatal proliferation of non-neuronal cells, developmental changes in gene expression patterns in various cell types, and the emergence of auditory activity. However, whether cell proximity plays a role in intercellular interaction and connectivity requires further study.

Our snRNA-seq and Stereo-seq data showed that all cell types could be identified at birth in both normal and congenital hearing loss mice, suggesting that the initial differentiation and diversification of CN cell types are independent of auditory activity. However, we found gene expression changes in a cell type in normal mice during postnatal development and in mutant mice. These changes may be associated with specific functional states of a cell type rather than the change of cell type. For example, GO analysis of markers in GBCs showed differences in functional enrichment. Thus, studying the various states of cell types will enhance our ability to distinguish core gene sets associated with specific functional states and further our understanding of the diverse functions of cell types as well as the biological basis of individual variability.

Auditory input can be disrupted by dysfunction or loss of peripheral cochlear synapses or spiral ganglion cells. Previous studies have shown that IHC malfunction can lead to degeneration of SGNs (loss of afferent fibers), as observed in *Vglut3*^*–/–*^ mice.^56,61^ However, it remains challenging to determine whether the auditory input-dependent changes observed in the CN are due primarily to neuronal activity deprivation or afferent fiber loss. Our findings suggest that reduced synaptic activity may play a more substantial role. This inference is supported by several lines of evidence. First, hearing restoration (via cochlear implant or gene therapy) in congenital hearing loss (mutations that could lead to afferent fiber loss) mouse models or patients can achieve better auditory outcomes.^56,74–76^ Second, our results showed no significant changes in CN neuronal proportions in the mutant mice. This finding may be explained by the convergent innervation pattern of CN neurons (e.g., bushy cells and fusiform cells), in which individual neurons receive input from multiple auditory nerve fibers. Thus, loss of a subset of these fibers may not critically affect neuronal survival, at least before P45. However, further experiments are needed to explicitly distinguish between the effects of neuronal activity deprivation and afferent fiber loss on the CN.

### *Spp1* is an auditory input-dependent gene involved in auditory processing

We identified *Spp1*^*+*^ bushy cells as the primary cell type affected by auditory nerve input. The appearance of *Spp1*^*+*^ cells apparently represents an auditory input-dependent transition of the cell state rather than the emergence of a new cell type, highlighting transcriptional regulation as a mechanism underlying activity-dependent neuronal plasticity in the CN. The *Spp1-*encoded OPN is a multifunctional glycophospho-protein involved in various physiological and pathological processes via an intracellular form (iOPN) and a secreted form (sOPN).^77^ Although most activities of OPN are ascribed to sOPN, accumulating evidence suggests that iOPN can perform specific functions in cell migration, motility, and immunity.^77–79^ For example, OPN remains localized in the cytoplasm of cortical pyramidal neurons without being secreted.^80,81^ Our snRNA-seq analyses of bushy cells in mice showed that *Spp1* deletion reduced the expression of genes associated with GO terms for neural development and synapse organization. This result is consistent with previous findings that *Spp1* can sensitize growth factor responses during regeneration of injured retinal ganglion cell axons and promote regrowth of corticospinal axons, as well as synapse reorganization and functional recovery, in both spinal cord and cortical injury mouse models.^82,83^

We observed that the interval between waves II and IV in auditory brainstem responses was significantly increased in *Spp1*^*–/–*^ mice, reflecting impaired auditory processing in the CN. The reduced amplitude of the sEPSCs could contribute to the delayed activation of action potentials in CN neurons, resulting in an increased latency in ABR waves. Two other mechanisms may also account for the ABR abnormality. First, as shown in Fig. 5, the expression of neurofilament genes (*Nefh*, *Nefm*, and *Nefl*), which are determinants of axonal diameter, was significantly reduced. This could decrease the conduction velocity of bushy cell axons.^84^ Second, our DEG analysis showed that gene expression in oligodendrocytes was affected by *Spp1* deletion, which led to changes in the expression of many proteins underlying myelination. Notably, gene expression in other glial cells (e.g., astrocytes) that could affect synaptic functions was also affected by *Spp1* deletion. This may result in a reduction of sEPSC amplitude, which in turn affects the latency of ABR waves. Taken together, our results show that *Spp1* is not only a useful molecular marker of a bushy cell subtype but also an important regulator of the expression of many proteins involved in auditory signal processing.

Increased wave latency may affect the temporal resolution of auditory signals, leading to impaired sound localization by interaural time differences, as at least some *Spp1*^+^ bushy cells project to the medial nucleus of the trapezoid body, which receives innervation from both ears.^85^ In addition, temporal cues are also important in speech recognition. Delayed activity due to *Spp1*^*–/–*^ neurons may impair speech discrimination by disrupting the timing of speech input to various higher-order nuclei in the auditory ascending pathway.^24,86^ Our findings demonstrate the important role of *Spp1* in auditory processing in the CN, although how auditory activity regulates *Spp1* expression in the CN remains to be elucidated.

### Clinical implications

Our findings have significant implications for hearing rehabilitation in patients with profound hearing loss. For early hearing restoration, previous studies have shown that cochlear implants in children under the age of 12 months result in better auditory, language, and adaptability outcomes than those in older patients.^35,36^ This finding may be attributed to the susceptibility of CN gene expression to auditory activity during the early postnatal period. It is consistent with the existence of critical periods during postnatal development when neural circuits are particularly sensitive to experience.^18^

Our findings also have important implications for the design and placement of auditory brainstem implant (ABI) electrodes. Notably, the Stereo-seq data indicated the predominant presence of *Spp1*^+^ bushy cells in the pAVCN, which may therefore be the most appropriate site for ABI stimulation. This inference is consistent with the finding of a CN electrical stimulation study in mice, which showed that the VCN could be the preferred site for the ABI array.^87^

### Limitations of the study

Although we present a comprehensive spatial transcriptome analysis of the CN, our data have several limitations. First, our description of cell types is based primarily on knowledge gained from previous physiological and histological studies. We used a conservative clustering approach that aimed to distinguish clearly distinct major cell types without further sub-clustering. Thus, heterogeneity could exist within the identified cell types. Second, we have not investigated the correspondence and potential relationship between transcriptomic signatures and specific morphologies or physiological functions of CN cells. Third, we used mouse models with full gene deletions, which differ from the missense mutations found in patients. Future research should also include other types of mutant mice, such as those with the *p.A221V* mutation in *Slc26a5*, or a mouse model with minimal cochlear cell death, to assess changes in CN cells associated with hearing loss. Fourth, since our deletion of *Spp1* was not specific to CN cells, we cannot exclude potential indirect mechanisms underlying the effect of *Spp1* deletion on *Spp1*^*+*^ bushy cell functions. Future studies in this regard may include the generation of animal models with cell-type-specific *Spp1* deletion, together with biochemical, histological, and physiological analyses, that could establish the relationship between *Spp1* expression and the function of bushy cells.

In conclusion, we have provided a valuable resource for understanding the molecular diversity, as well as the composition and distribution of cell types, in the mouse CN. In addition, we have investigated changes in gene expression and the spatial organization of CN cells in normal mice during postnatal development and in mutant mice with congenital hearing loss, and we have demonstrated that bushy cells expressing *Spp1* are the primary cell type that exhibits hearing loss-induced alterations in gene expression. Overall, our data provide a valuable resource for future studies of the physiology and pathophysiology of auditory processing in the CN.

## Materials and methods

### Animals

WT (C57BL/6J) and mutant (C57BL/6J background) mice of both sexes were used for the experiments. WT mice were obtained from Shanghai Model Organisms Center (Shanghai, China). *Otof*^*–/–*^ and *Vglut3*^*–/–*^ mice were generated in our previous studies on a C57BL/6J background, and their knockout information and auditory phenotypes were described in our previous publications.^56,88^ *Ush1c*^*–/–*^ mice were purchased from GemPharmatech (Nanjing, China; #T027998), with exons 2–8 knocked out on a C57BL/6J background.

For the *Vglut3*^*–/–*^+GT mice (the mutant mice were treated with gene therapy at about 3 weeks of age and sacrificed 3 weeks later), we used *Vglut3*-3× Flag packaged in the adeno-associated virus 8 (AAV8) delivery vector driven by the CMV promoter to restore VGluT3 expression. The method of inner ear injection was described in our previous publications.^56,89,90^ In brief, mice were anesthetized with an intraperitoneal injection of xylazine (10 mg/kg) and ketamine (100 mg/kg). AAV8-CMV-*Vglut3*-3× Flag (total volume of 1000 nL) was injected into the inner ears through the posterior semicircular canal at a rate of 169 nL/min using glass micropipettes on the Nanoliter Microinjection System (WPI, USA). After injection, the skin incision was closed using veterinary tissue adhesive (Millpledge Ltd., UK). The mice were placed on a 42 °C heating pad for recovery. Standard postoperative care was applied after surgery.

Mice were housed throughout the experiments in the animal care facility of the Ear Institute of Shanghai Ninth People’s Hospital, affiliated with Shanghai Jiao Tong University School of Medicine. Mice were maintained on a standard 12-h light/dark cycle with an ambient temperature of 22–25 °C and humidity of 50%–60%. The experimental protocol was approved by the Institutional Animal Care and Use Committee of Shanghai Ninth People’s Hospital (SH9H-2022-A926-1) and followed the guidelines for the Care and Use of Laboratory Animals (8th edition) published by the National Institutes of Health (Bethesda, MD, USA). Prior to tissue collection, the mice were euthanized by administration of an isoflurane (RWD, China) overdose via inhalation. Age- and sex-matched littermates were used for subsequent comparative studies of WT and mutant mice at the age of P45.

### Stereo-seq library preparation and sequencing

*Tissue processing* The dissected CN was processed as described in a previous study.^37^ In brief, the CN was placed on a glass slide, rapidly frozen with dry ice, and then placed in an optimal cutting temperature (OCT) cryomold. Before loading the sections onto the Stereo-seq chip, we examined the CN morphology of the collected sections under a microscope. Cryosections were cut at a thickness of 10 μm using a Leica CM1950 cryostat. For CN #1 (animal 1, male), 15 consecutive sagittal sections were collected on the basis of appropriate morphology. For CN #2–#5 (animals 2–5, male), 4 sagittal sections were collected from each animal at 60-μm intervals (equivalent to skipping 6 sections between collections) with different starting coordinates. For CN #6 (animal 6, male), 4 coronal sections were collected from one mouse at 120-μm intervals (skipping 12 sections between collections). Tissue sections were adhered to the Stereo-seq chip, incubated at 37 °C for 3–5 min, and then fixed in methanol for ~40 min. Nucleic acid dye (Thermo Fisher Scientific, Q10212) was used to stain the sections for visualization of single-stranded DNA (ssDNA). Images of the stained CNs were captured using a Motic Custom PA53 FS6 microscope prior to in situ capture of fluorescein isothiocyanate signals (objective 10×).

*In situ reverse transcription, amplification, library construction and sequencing* We washed tissue sections using 0.1× SSC buffer (BBI, B548109-0200) supplemented with 0.05 U/μL RNase inhibitor (NEB, M0314L). Tissue sections placed on the chip were permeabilized with PR Enzyme (STOmics Gene Expression kit S1) in 0.01 M HCl buffer at 37 °C for 12 min, and then washed with 0.1× SSC buffer supplemented with 0.05 U/μL RNase inhibitor. mRNAs captured by the DNBs were reverse transcribed using SuperScript II Reverse Transcription mix (STOmics Gene Expression Kit S1, containing RT Reagent, RT Additive, RT Oligo, ReverseT Enzyme, and 0.05 U/μL RNase inhibitor) at 42 °C for 1 h.

After reverse transcription, cDNAs on the chip were amplified using KAPA HiFi HotStart Ready Mix (Roche, KK2602) with 0.8 μM cDNA-PCR primers. Sequencing libraries were prepared from PCR products (20 ng DNA) by fragmentation (in-house Tn5 transposase), amplification (KAPA HiFi HotStart Ready Mix), and purification (VAHTS DNA Clean Beads). The final libraries were sequenced on an MGI DNBSEQ-T7 sequencer.

### Preparation of single-nucleus suspensions and snRNA-seq

The dissected tissue was transferred to a pre-chilled 2-mL tissue Dounce homogenizer (Sigma, #D8938-1SET) containing 2 mL of ice-cold homogenization buffer. This buffer contained 500 mM sucrose (Sigma, #69293), 1% BSA (Sigma, #V900933-100G) in nuclease-free water, 20 mM Tris, pH 8.0 (Sigma, #T2694-1L), 50 mM KCl (Sigma, #P5405), 10 mM MgCl_2_ (Sigma, #2670-100 g), 0.1% NP-40 (Invitrogen, #FNN0021), 1× protease inhibitor cocktail (Thermo Fisher Scientific, #87786), 0.1 mM DTT (Sigma, #646563), and 0.12 U/µL RNasin Plus (Promega, #N2115). After incubation on ice for 5 min, the mixture was homogenized with 25 strokes of the loose Dounce pestle (pestle A). The homogenate was then filtered through a 70-µm strainer, further homogenized with 25 strokes of the tight pestle (pestle B), and filtered through a 40-µm strainer into a 15-mL centrifuge tube to pellet the nuclei. The pellet was resuspended in 1.5 mL of blocking buffer containing 1× phosphate-buffered saline (PBS, Thermo Fisher Scientific, #10010049), 1% filter-sterilized BSA, and 0.2 U/mL RNasin Plus (Promega, #N2115). Resuspension was achieved by gentle up-and-down pipetting, followed by centrifugation at 500× *g* for 5 min. The nuclei were then resuspended in cell resuspension buffer, maintaining a concentration of at least 1000 nuclei/μL for library preparation.

The DNBelab C Series High-throughput Single-Cell RNA Library Preparation Kit (MGI, 940-000047-00) was used to prepare snRNA-seq libraries as described previously.^91^ In brief, single-nucleus suspensions were loaded onto a chip to generate droplets, which were then incubated at room temperature for 20 min to facilitate capture of mRNA released from the cells. Emulsion breakage and bead collection were performed, followed by reverse transcription, cDNA amplification, and purification. The PCR products were used to generate DNBs and subsequently sequenced on an MGI DNBSEQ-T1 or T7 platform at BGI Qingdao (Qingdao, China). The sequencing strategy used was a 41-bp read length for Read 1 and a 100-bp read length for Read 2.

### Stereo-seq data analysis

*Processing of raw Stereo-seq data* The raw Stereo-seq data were processed as described previously.^37^ Read 1 contains coordinate identity (CID, 1–25 bp) and molecular identifier (MID, 26–35 bp), and Read 2 consists of the cDNA sequences. In brief, the CID sequences were first aligned to the designated coordinates on the chip, allowing for a 1-base mismatch. UMI sequences with a quality score lower than 10 were subsequently removed. The CID and MID associated with each read were appended to each read header. Retained reads were then aligned to the reference genome (GRCm38/mm10, https://genome-asia.ucsc.edu/cgi-bin/hgGateway?redirect=manual&source=genome.ucsc.edu) using STAR (v2.7.9a). Reads with mapping quality (MAPQ) > 10 that were annotated to genes were counted. UMIs with the same CID and the same gene locus were collapsed, allowing 1 mismatch to be corrected for sequencing and PCR errors. The full protocol is available via the SAW pipeline at https://github.com/BGIResearch/SAW.

*Spatial clustering of Stereo-seq data* The spatial expression profile matrix of the CN was divided into bins of size 50 (50 DNBs), as well as bins of sizes 15, 30, 100, and 200. Transcripts of the same gene were aggregated within each bin. Unsupervised clustering was then performed on datasets with different bin sizes (i.e., BIN15, BIN30, BIN50, BIN100, and BIN200). The Seurat NormalizeData function was used to normalize the data, and FindVariableFeatures was used to identify 2000 highly variable genes for creating the SpatialExperiment object. Following the recommendations of BANKSY,^39^ a lambda of 0.8 and a k_geom of 30 were used in the computeBanksy function to facilitate domain segmentation. Dimensionality reduction was performed using the runBanksyPCA function at the sample level, and batch effects across chips were corrected using Harmony (v0.1.1).^92^ The post-Harmony embedding was then used to compute UMAP through the runBanksyUMAP function, which enabled spatial clustering at a resolution of 1.5 using the clusterBanksy function. Sporadic points within the domain were eliminated using the smoothing method implemented in smoothLabels. Finally, the FindAllMarkers function in Seurat (v4.3.0) was used to identify spatial region-specific variable genes (SVGs). The optimal number of spatial regions and their annotations were determined on the basis of anatomical annotations reported in previous studies, as well as the SVGs. Since the other clusters could not form a specific spatial region, such as the astrocytes, we did not analyze them further.

*Image-based single-cell segmentation and unsupervised clustering* To achieve single-cell resolution for the Stereo-seq data, we defined the boundary of each cell on the basis of the ssDNA image. We registered the DNB image with the nucleic acid-stained image and performed manual cell segmentation using StereoCell (v1.1.0).^93^ The UMIs contained in each nucleus were directly assigned to each cell. Furthermore, to retrieve the UMIs in the “cytoplasm”, we used a Gaussian mixture model to estimate the probability of each remaining UMI belonging to a given cell based on the initial nuclei segmentation. Finally, we determined the centroid of each segmented cell using a convex hull algorithm. We aggregated the UMIs that belonged to the same cell for each gene and generated a cell–gene matrix for downstream analysis.

We refined our analysis by filtering out genes expressed in fewer than three segmented cells and excluding genes associated with sex determination and stress responses, such as *Xist*, *Tsix*, *Eif2s3y*, *Ddx3y*, *Uty*, *Kdm5d*, *Egr1*, and *Jun*. Segmented cells with fewer than 200 detected genes were removed.

The segmented cells that passed our filters were further processed using a suite of Seurat (v4.3.0) functions, including NormalizeData, FindVariableFeatures, FindIntegrationAnchors, IntegrateData, ScaleData, RunUMAP, FindNeighbors, and FindClusters.

Finally, we annotated the resulting cell clusters on the basis of marker genes identified during the cell type identification process in our snRNA-seq data. Clusters with identical annotations were consolidated to provide a coherent representation of the cellular landscape within the CN.

*Replicability of cell types across Stereo-seq and snRNA-seq data* We used the MetaNeighbor algorithm^94^ to quantify the replicability of cell types across the Stereo-seq and snRNA-seq data. In brief, we merged the datasets using the mergeSCE() function and identified the highly variable gene set using the variableGenes() function with default parameters. We then generated the AUROC_matrix using the MetaNeighborUS() function.

*Analysis of cell-type composition across the CN* (1) Spatial module analysis: we used BANKSY software to identify spatial modules using Stereo-seq-defined cell types. The procedure was similar to the spatial region analysis described above. (2) Cell–cell proximity analysis: for each cell, we first identified the nearest 30 neighbors on the basis of spatial distance. We then determined the cell types of these neighboring cells and obtained the cell-type composition for the queried cell. After processing all cells within the given cell types, we calculated the frequency of paired cell–cell occurrences to generate a cell–cell proximity matrix. To compare cell proximity among different groups, we calculated the normalized cell–cell proximity matrix by taking the log_2_ ratio of the “Group A” matrix to the “Group B” matrix. We then performed Wilcoxon rank tests using the Wilcoxon.test function in R and adjusted the resulting *P*-values using the p.adjust function in R with the BH method for multiple comparisons.^95^ (3) Cell-interaction score analysis: the normalized cell-interaction score of the aAVCN and pAVCN was calculated using sq.gr.interaction_matrix in Squidpy. To visualize the cell proximity between the aAVCN and pAVCN, we used the R package ComplexHeatmap. (4) Cell-type niche analysis: we examined the cell-type composition in an area of BIN100 (50 μm × 50 μm) as described in a previous study.^96^ We then constructed a cell-type matrix and created a Seurat object. A cumulative cell-composition ratio > 0.16 was used for subsequent clustering analysis in Seurat (v4.3.0) with a resolution parameter of 0.05. To determine the dominant cell type and cell-composition differences in each niche, a Wilcoxon test between an individual niche and other niches was used.

*Spatial gene expression in the CN* To compare the spatial expression of specific genes, we implemented the following procedure. First, we organized the tissue samples into distinct arrangements, specifically left and right (anterior and posterior) as well as upper and lower (dorsal and ventral). Next, the expression level of the marker gene in each tissue sample was normalized to a range of 0–1 to allow comparison. Then, to ensure robustness in the analysis, we eliminated the top 10% of outliers based on the scaled gene-expression values. Finally, we used the geom_smooth function in ggplot2 (v4.2.2) to generate a fitted curve for the gene expression. The confidence interval for the fitted curve was set to 0.75.

### snRNA-seq data analysis

*Processing of raw snRNA-seq data* Raw sequencing data were filtered and demultiplexed using PISA software (v0.7, https://github.com/shiquan/PISA). The remaining reads were then aligned to the reference genome (GRCm38/ mm10) using STAR (v2.7.9a) and sorted by Sambamba (v0.7.0). For snRNA-seq data, the annotation parameter “-intron” was added to count reads that matched introns or exons as transcripts of each gene. Finally, PISA was used to generate the gene expression matrix for downstream analysis.

*Clustering and marker gene identification* We first used the DoubletFinder package to remove doublets for each library. Basic processing and visualization of the snRNA-seq data were performed using the Seurat package (v4.3.0) in R (v4.2.2). We excluded cells with a number of genes (nFeature) less than 200 or greater than 6000, as well as cells with a percentage of mitochondrial genes (percent.mt) > 5%. To minimize potential noise, genes detected in fewer than 3 cells were also removed. Sex- and stress-related genes, including *Xist*, *Tsix*, *Eif2s3y*, *Ddx3y*, *Uty*, *Kdm5d*, *Egr1*, and *Jun*, were also excluded from the analysis. The filtered data were then normalized using the NormalizeData function with a default factor of 10,000. We selected 2000 highly variable genes on the basis of their average expression and dispersion level. For each function, we manually adjusted the parameters to achieve optimal clustering results. Integration anchors were identified using the FindIntegrationAnchors function. Data integration was performed using the IntegrateData function, and gene expression was centered using the ScaleData function. Principal component analysis (PCA) was performed using the RunPCA function with 60 principal components. UMAP dimensionality reduction was performed using the RunUMAP function with 60 principal components. After PCA, we selected the appropriate principal components for clustering with a resolution of 0.5. We assigned cell types to the clusters according to the expression of canonical markers.

*Prediction of snRNA-seq-defined cell clusters* Decomposition was performed with RCTD^59^ to predict the locations of snRNA-seq-defined cell clusters. We first constructed a reference using the snRNA-seq dataset and then used the RCTD package to estimate the mixture and cell cluster identities at the BIN20 resolution of the Stereo-seq data by fitting a statistical model. We calculated the weight of the cell clusters and filtered values with a threshold of 0.3.

*Identification of auditory input-associated GEPs* To investigate the response of CN cells to acoustic signal stimulation, we used the cNMF (v1.4) implementation of consensus non-negative matrix factorization (https://github.com/codyheiser/cnmf). Raw count matrices from WT and mutant mice were combined into a single AnnData object and used as input. Batch effects were corrected using Harmony, following the recommendations in the cNMF documentation. Non-negative matrix factorization was performed using the cNMF factorize command with 100 iterations. This procedure was repeated 30 times to derive consensus NMF components, each corresponding to a GEP. To select the appropriate number of GEPs, we evaluated models with *k* ranging from 10 to 40 and chose the optimal *k* by jointly considering reconstruction error and solution stability. Cell usage values were normalized such that the total usage per cell summed to 1. Cells were considered to express a given GEP if the corresponding usage exceeded 0.25. For phenotype comparisons, GEP usage — reflecting cellular state — showed consistent shifts across all healthy vs mutant comparisons.

*Identification of differential network genes by cell-specific network (CSN) analysis* We used the locCSN (v0.0.12) package^97^ to investigate the CSNs of the snRNA-seq data. First, we used MetaCell^98^ to generate metacells by partitioning bushy cells into small and cohesive groups within each comparison on the basis of their expression profile similarity. For each metacell, gene expression was calculated as the average counts per million of all constituent cells, followed by a log_2_ transformation. We used the NumPy linalg. norm function to identify the top 100 nearest metacells. We then invoked locCSN.csn_block_loc to estimate the CSN test statistics for bushy CSNs between WT and mutant mice. We then used the norm.ppf function with a parameter of 0.99 and set the CSN test statistic threshold to *α* = 0.01 to extract the most noteworthy interactions. Finally, differential network genes were identified by averaging the CSN test statistics between WT and mutant mice with significance testing using the sLED method implemented in the locCSN R code.

*Analysis of DEGs* We performed DEG analysis using the FindMarkers or FindAllMarkers function in Seurat (v4.3.0). DEGs were defined as genes with a log_2_(fold change) > 0.1 and adjusted *P* ≤ 0.05.

We performed a case-control analysis using the Cacoa package^64^ to compare mutant mice with WT mice. Expression shift was analyzed using the normalized expression distance for each snRNA-seq-defined cell type. All models were analyzed using the default parameters. Statistical analysis was performed according to the package’s methods.

We analyzed gene expression patterns during CN development using the ImpulseDE2 package.^99^ Temporal DEGs were identified by time-series analysis of read counts using ImpulseDE2 with an adjusted *P* ≤ 0.05. Finally, we used a monotonous sigmoid model in the package to classify the DEGs into four patterns.

For the identified DEGs, we used the irGSEA (integration of single-cell rank-based gene set enrichment analysis) R package^62^ to associate them with each cell type. We used the SingScore scoring method to evaluate the enrichment score across different cell types.

*Gene module identification* We determined gene modules using the Hotspot package (v0.9.0). We used the single-cell expression matrix of identified DEGs obtained from the comparison analysis or time-series analysis. Then, using the Hotspot function, we identified fitness-related genes that were significantly autocorrelated using the “danb” observation model and 15 neighbors. Genes with an FDR < 0.05 were retained for further analysis. We then computed pairwise local autocorrelations and clustered genes using these pairwise statistics with the “Create Modules” function in Hotspot, setting an appropriate minimum gene threshold.

### Gene regulatory network analysis

We constructed a gene regulatory network for bushy cells using the pySCENIC (v0.12.1) pipeline (a scalable SCENIC workflow for single-cell gene regulatory network analysis). In brief, we used “pyscenic grn” for network inference between TFs and targets, performed regulon prediction using “pyscenic ctx”, and calculated and binarized the activity of each TF in single cells with the AUCell function of pySCENIC. To identify the main TFs in bushy cells, we used the RSS method implemented in the calcRSS function in the SCENIC R package, which has been shown to be an effective method for the identification of cell type-specific TFs.

### snATAC-seq data analysis

*snATAC-seq library construction and sequencing* Sequencing libraries were constructed using the DNBelab C Series Single-Cell ATAC Library Prep Set (MGI, 1000021878) in accordance with the manufacturer’s instructions. In brief, single nuclei were isolated by grinding flash-frozen dissected tissue in lysis buffer with a 2-mL homogenizer (Sigma, #D8938-1SET). The nuclei were then washed twice with wash buffer and resuspended in 100 μL of the same buffer. After counting, 50,000 nuclei were aliquoted and treated with in-house Tn5 transposase. The transposed single-nucleus suspensions were converted into barcoded snATAC-seq libraries through droplet encapsulation, pre-amplification, emulsion breakup, bead capture, DNA amplification, and purification. Indexed sequencing libraries were prepared following the manufacturer’s protocol, quantified with the Qubit ssDNA Assay Kit, and sequenced on an MGI DNBSEQ-T7 instrument.

*Processing of raw snATAC-seq data* Raw sequencing reads were demultiplexed and aligned to the GRCm38 (mm10) reference genome using DNBelab C Series HT scRNA analysis software (https://github.com/MGI-tech-bioinformatics/DNBelab_C_Series_HT_scRNA-analysis-software). A count matrix and fragment files were then generated for nuclear RNA analysis. To integrate snATAC-seq datasets across all libraries, we created a unified set of non-overlapping peaks by merging intersecting peaks using the reduce function from the GenomicRanges package. Read counts within these consensus peaks were then quantified per cell to construct Signac objects for each library (https://stuartlab.org/signac). Subsequent quality control was performed with Signac (v1.14.0). The TSSEnrichment and NucleosomeSignal functions were applied to calculate transcription start site (TSS) enrichment scores and nucleosome signals, respectively. Only cells with a TSS enrichment score > 2 were retained for downstream analyses.

*snATAC-seq data clustering and annotation* We merged all the Signac objects and annotated the resulting data using the mouse GRCm38 genome assembly. The integrated dataset was then subjected to TF-IDF normalization, followed by dimensionality reduction by latent semantic indexing, UMAP projection, and clustering. To annotate the snATAC-seq data, we used our snRNA-seq data as a reference. First, a Seurat object was created from the gene-score matrix derived from the snATAC-seq data. Integration was performed using the CCA method via the FindTransferAnchors function, resulting in a transfer.anchors object. The snATAC-seq data were then projected onto the reference UMAP, together with transferred cell-type labels, using the MapQuery function. Peaks with a predicted cell-type score > 0.8 were retained for subsequent analysis.

*Cell type-specific accessibility peaks and motif enrichment analysis* To identify differentially accessible peaks in bushy cells compared with other cell types, we used the FindMarkers function. We used a log_2_(fold change) threshold of 0.25 and Bonferroni-adjusted *P*-values to assess the significance of differentially accessible peaks. Subsequently, motif enrichment analysis was performed on these specific differential peaks based on analysis with the JASPAR2020 dataset. Specifically, the GC content (i.e., the proportion of guanine and cytosine nucleotides) was calculated for each differential peak. A background set of 40,000 peaks was then selected such that it closely matched the overall GC content, accessibility, and width profile of the differential peaks. This matching process was carried out using the FindMotifs function in Signac. From the resulting motifs, we focused on those associated with differential peaks located within the *Spp1* genomic region.

### Immunohistochemistry and confocal microscopy

Animals were perfused with ice-cold PBS followed by ice-cold 4% paraformaldehyde after deep anesthesia. Tissues were dissected from P1–P45 mice and post-fixed in paraformaldehyde at 4 °C for 1 h. The samples were then washed in PBS for 30 min, dehydrated in 30% sucrose for 2 h at 4 °C, and embedded in OCT compound (Tissue-Tek). Tissue sections were cut at a thickness of 20 µm using a Leica cryostat (Leica, Germany, #CM3050S), permeabilized, and blocked in 1% (v/v) Triton X-100 and 5% (w/v) BSA/PBS at room temperature for 60 min before incubation with primary antibodies: rabbit anti-GABA A receptor α6 (Abcam, UK, #ab300069, 1:200), rabbit anti-carbonic anhydrase VIII (Proteintech, USA, #12391-1-AP, 1:200), rabbit anti-somatostatin 28 (Abcam, UK, #ab111912, 1:200), mouse anti-SPP1 (Santa Cruz, USA, #sc-21742, 1:200), goat anti-SPP1 (Abcam, USA, #ab11503, 1:200), mouse anti-calretinin (Merck-Millipore, Germany, #MAB1568, 1:500), rabbit anti-calretinin (Abcam, UK, #ab92341, 1:500), mouse anti-FOXJ1 IgG1 (Invitrogen, USA, #14-9965-95, 1:200), rabbit anti-TAFA1 (Atlas Antibodies, Sweden, #HPA013407, 1:100), rabbit anti-ATOH7 (Novus, USA, #NBP1-88639, 1:200), rabbit anti-HHIP (Sigma-Aldrich, USA, #SAB2702069, 1:200), rabbit anti-PENK (LSBio, USA, #LS-B15645, 1:200), rabbit anti-NEFH (Proteintech, USA, #18934-1-AP, 1:200), anti-tubulin β3 IgG2b (BioLegend, USA, #801201, 1:500), rabbit anti-MAFB (Merck-Millipore, Germany, #ABE55, 1:200), guinea pig anti-VGluT1 (Synaptic Systems, Germany, #135304, 1:500), rabbit anti-NeuN (Abcam, UK, #ab177487, 1:500), mouse anti-IBA1 (Abcam, UK, #ab283319, 1:500), rabbit anti-SPARCL1 (Invitrogen, USA, #PA5-80063, 1:200), and mouse anti-GluR2 IgG2a (Merck-Millipore, USA, #MAB397, 1:200).

The secondary antibodies used were Alexa Fluor 488-conjugated goat anti-rabbit IgG (Invitrogen, USA, #A-11008, 1:500), Alexa Fluor 555-conjugated goat anti-mouse IgG1 (Invitrogen, USA, #A-21127, 1:500), Alexa Fluor 647-conjugated goat anti-mouse IgG2a (Invitrogen, USA, #A-21241, 1:500), Alexa Fluor 647-conjugated goat anti-guinea pig IgG (Invitrogen, USA, #A-21450, 1:500), Alexa Fluor 555-conjugated donkey anti-goat IgG (Invitrogen, USA, #A-21432, 1:500), Alexa Fluor 488-conjugated donkey anti-rabbit IgG (Invitrogen, USA, #A-21206, 1:500), and Alexa Fluor 647-conjugated donkey anti-mouse IgG (Invitrogen, USA, #A-32787, 1:500). Finally, DAPI (Invitrogen, USA) was applied to each slide for 10 min at room temperature. The slides were then covered with a microscope cover slip (Fisher Scientific, Germany). Confocal images were acquired using a Leica TCS SP8 microscope and processed using Imaris software (v9.2.0). To measure the volume proportion of type Ia and non-type Ia auditory endbulbs of Held synapses, images were acquired using a 60× oil immersion objective with a *z*-step size of 0.3 μm and a resolution of 1024 × 1024 pixels. Three-dimensional reconstructions of VGluT1-labeled puncta (blue) and type Ia endbulbs of Held terminals (red) in the target bushy neuron were created using Imaris, as described in our previous study.^65^ VGluT1-labeled puncta located inside type Ia endbulbs were determined as puncta from type Ia synapses and shown in pink (overlap of red and blue), whereas those outside type Ia endbulbs were from non-type Ia synapses and remained blue. The volumes of VGluT1/CR-labeled puncta were measured using Imaris software.

### Single-molecule fluorescence in situ hybridization (smFISH)

To generate an antisense RNA probe, a target DNA fragment (Supplementary information, Table S6) was synthesized from the pUC57 plasmid (Generay Biotechnology, China). Antisense probes labeled with digoxigenin (DIG) were prepared using the T7 RNA Polymerase kit (Promega, USA, #P2075) and the DIG RNA Labeling kit (Roche, Switzerland, #11277073910).

The CN was dissected and fixed in DEPC-PFA at 4 °C for 1 h, and then washed in DEPC-PBS for 30 min and dehydrated in 30% DEPC-sucrose at 4 °C for 2 h. Next, 12-µm-thick tissue sections were prepared using a Leica cryostat (Leica, Germany, #CM3050S). These sections were dried at 37 °C for 10 min, fixed in 4% DEPC-PFA at room temperature for 15 min, and washed with DEPC-PBS at room temperature for 3 min. Endogenous peroxidase was removed by treating all sections with 0.2% H_2_O_2_ at room temperature for 30 min (repeated twice). The sections were then washed in DEPC-PBS for 3 min and incubated in 0.2 M HCl at room temperature for 10 min. After an additional wash with PBS for 3 min, the sections were incubated in 0.1 M TA-HCl buffer consisting of 662.5 µL triethanolamine (Sigma-Aldrich, USA, #1371481000) and 1.35 mL 1 M HCl, and the final volume was adjusted to 50 mL with DEPC-H_2_O, pH 8.0, at room temperature for 1 min. Samples were then incubated in 0.1 M TA-HCl buffer containing 0.25% acetic anhydride for 10 min at room temperature.

The samples were dehydrated with a series of ethanol concentrations: 60%, 80%, 95%, and 100% (twice) at room temperature for 90 s each, and then incubated in hybridization buffer at 60 °C overnight. The next day, the slides were washed sequentially with 5× SSC, 2× SSC with 50% formamide, and TNE buffer with 20 µg/mL RNase (Takara, Japan, #2158) at 37 °C for 30 min. Subsequently, the samples were washed with 2× SSC at 60 °C for 20 min, 0.2× SSC at 60 °C for 20 min, and 0.1× SSC at room temperature for 20 min, and then blocked with 0.5% blocking reagent (Roche, USA, #11096176001) at room temperature for 1 h. Finally, the sections were incubated with anti-DIG-POD (Roche, USA, #11207733910, 1:500) at 4 °C overnight. On the third day, the signal was detected using a TSA Cyanine 3 System (Akoya Biosciences, USA, NEL741001KT).

### Western blot analysis

After animals were deeply anesthetized and sacrificed, the VCN was immediately dissected in ice-cold PBS. For each sample, 4–6 VCNs were collected, pooled, and treated with ice-cold radioimmunoprecipitation assay lysis buffer containing protease inhibitor cocktail and phosphatase inhibitors (Thermo Fisher Scientific, USA, #78440). The samples were centrifuged at 13,000× *g* for 20 min at 4 °C, the supernatants were collected, and the total protein concentration was determined using a BCA Protein Assay Kit (Beyotime Institute of Biotechnology, China, #P0010). Equal amounts of total protein were loaded onto 8% Bis-Tris gels (GenScript, China, #M00663).

After electrophoresis at 80 V for 90 min, the proteins were transferred to a PVDF membrane (Beyotime Institute of Biotechnology, China, #FFP19) at 400 mA for 35 min. After blocking with blocking solution (Beyotime Institute of Biotechnology, China, #P0023B) for 30 min at room temperature, the membranes were successively incubated with the following antibodies overnight at 4 °C: rabbit anti-NEFH (Proteintech, USA, #18934-1-AP, 1:1000), rabbit anti-SPARCL1 (Invitrogen, USA, #PA5-80063, 1:1000), mouse anti-GluR2 (Sigma-Aldrich, USA, #MAB397, 1:1000), and rabbit anti-GAPDH (Cell Signaling Technology, USA, #3683, 1:1000). Blots were then incubated with HRP-conjugated secondary antibodies for 1 h at room temperature: peroxidase-labeled goat anti-rabbit IgG (Abcam, UK, #ab6721, 1:10,000) and peroxidase-labeled goat anti-mouse IgG (Abcam, UK, #ab205719, 1:10,000).

After extensive washing of the membrane, the protein bands were visualized with an Amersham Imager 600 (GE Healthcare, Little Chalfont, UK) using an enhanced chemiluminescence procedure. ImageJ software (National Institutes of Health, Bethesda, MD, USA) was used to calculate the relative densities of the target proteins.

### Generation of *Spp1*-knockout mice

The *Spp1*-knockout mouse (C57BL/6J background) was designed and developed by GemPharmatech (Nanjing, China). The experimental procedure for generating the animal model was as follows: *Cas9* mRNA was synthesized in vitro using the mMESSAGE mMACHINE T7 Ultra Kit (Invitrogen, USA, #AMB13455) according to the manufacturer’s instructions. Two guide RNAs (gRNAs) targeting the deletion of exons 4–7 were also generated by in vitro transcription using the MEGAscript T7 Kit (Invitrogen, USA, #AM1354). These gRNAs were synthesized by Sangon Biotech (Shanghai, China), and their sequences were as follows: 5′-AACATGTAAAGGTACTGGTT-3′ and 5′-ACAGACACAAGGTTCTATTA-3′. After synthesis, the in vitro-transcribed gRNAs were purified using the NucleoSpin miRNA Kit (Macherey-Nagel, Germany), and their amounts were determined using a NanoDrop spectrophotometer. A mixture containing each gRNA at a concentration of 30 ng/μL and *Cas9* mRNA at a concentration of 110 ng/μL (TriLink Biotechnologies, USA) was then injected into zygotes, which were implanted into pseudopregnant females. The resulting offspring, known as founders, were screened for mutations in the targeted exons and for the presence of large deletions in the intervening sequences. Once confirmed, the founders were backcrossed to C57BL/6J mice and subsequently bred to propagate the animal model for use in the experiments described here.

### Hearing assessment

Mice were anesthetized by a combined intraperitoneal injection of ketamine (30 mg/kg; Sigma-Aldrich, USA) and pentobarbital (50 mg/kg; Sigma-Aldrich, USA). ABR recordings were performed in a sound-attenuating chamber. We used a TDT RZ6 workstation (Tucker–Davis Technologies, USA) to deliver acoustic stimuli and record the corresponding response signal. Short-tone burst stimuli with a duration of 3 ms and a rise/fall time of 1 ms were presented in a free field through an MF-1 speaker positioned 10 cm from the vertex at a rate of 20 stimuli/s. Stimulus frequencies ranged from 32.0 kHz to 4.0 kHz and were presented in half-octave steps. For each frequency measured, the sound level started at 90 dB SPL and then decreased in 5-dB steps until it reached two levels below the visible threshold. Each waveform was averaged over 400 trials. The latency (ms) and amplitudes (μV) of ABR wave I were analyzed offline using BioSigRZ software (v5.1, Tucker–Davis Technologies, USA).

### Brain slice preparation and electrophysiological recording

After mice (P20–P24) were deeply anesthetized and decapitated, their skulls were opened to remove the brainstem. Parasagittal slices containing the CN were cut at a 200-μm thickness using a VT1200S microtome (Leica Biosystems). Tissues were dissected and sectioned in artificial cerebrospinal fluid (ACSF) at 34 °C. The ACSF contained 122 mM NaCl, 3 mM KCl, 1.25 mM NaH_2_PO_4_, 25 mM NaHCO_3_, 20 mM glucose, 3 mM myo-inositol, 2 mM sodium pyruvate, 0.4 mM ascorbic acid, 1.8 mM CaCl_2_, and 1.5 mM MgSO_4_ and was gassed with 95% O_2_ and 5% CO_2_. Slices were incubated in the same ACSF at 34 °C for 30–45 min before recording began.

After incubation, brain slices were transferred to a recording chamber under a microscope (Axio Examiner, Carl Zeiss) and bathed in the same ACSF continuously flowing at a rate of 2–3 mL/min. Whole-cell recording was performed using an EPC10/2 amplifier (HEKA Electronics, Germany) driven by PATCHMASTER software (HEKA Electronics, Germany). Patch pipettes were pulled from borosilicate glass capillaries (World Precision Instruments, USA) using a vertical puller (PC-100, Narishige, Japan) to obtain a resistance range of 6–7 mΩ and filled with electrode solution containing 126 mM potassium gluconate, 6 mM KCl, 2 mM NaCl, 10 mM HEPES, 0.2 mM EGTA, 4 mM MgATP, 0.3 mM GTP, and 10 mM Tris-phosphocreatine, with the pH adjusted to 7.20. All chemicals were purchased from Sigma-Aldrich (USA). The electrode solution also contained 0.5% Neurobiotin-488 (Vector Laboratories, UK) or 0.5% biocytin (Sigma-Aldrich, USA) to fill the recorded neurons for visualization (stained with streptavidin-Alexa Fluor 488; Thermo Fisher Scientific, USA). Igor Pro (WaveMetrics Inc., USA) was used for data analysis.

Fixed brain slices with dye-filled neurons were processed for immunostaining as described previously. The primary antibodies used were rabbit anti-somatostatin 28 (Abcam, UK, #ab111912, 1:200), mouse anti-SPP1 (Santa Cruz, USA, #sc-21742, 1:200), rabbit anti-ATOH7 (Novus, USA, #NBP1-88639, 1:200), rabbit anti-HHIP (Sigma-Aldrich, USA, #SAB2702069, 1:200), rabbit anti-TAFA1 (Atlas Antibodies, Sweden, #HPA013407, 1:100), rabbit anti-PENK (LSBio, USA, #LS-B15645, 1:100), and rabbit anti-C1QL1 (Biorbyt, USA, #orb1260, 1:100).

### Data analysis and statistical tests for animal experiments

GraphPad Prism (GraphPad Software Inc., USA) was used for statistical analysis. Depending on the nature of the data set, statistical significance was assessed using a two-tailed unpaired Student’s *t*-test, Mann–Whitney *U* test, or one- or two-way ANOVA followed by a Bonferroni post hoc test. Results are presented as mean ± SD, and the significance level was set at *P* < 0.05. Significant differences are indicated as *P < 0.05, **P < 0.01, and ***P < 0.001.

## Supplementary information

Supplementary information, Figure S1

Supplementary information, Figure S2

Supplementary information, Figure S3

Supplementary information, Figure S4

Supplementary information, Figure S5

Supplementary information, Figure S6

Supplementary information, Figure S7

Supplementary information, Figure S8

Supplementary information, Figure S9

Supplementary information, Figure S10

Supplementary information, Figure S11

Supplementary information, Figure S12

Supplementary information, Figure S13

Supplementary information, Figure S14

Supplementary information, Table S1

Supplementary information, Table S2

Supplementary information, Table S3

Supplementary information, Table S4

Supplementary information, Table S5

Supplementary information, Table S6

Supplementary information, Data S1

## Data Availability

The processed data can be accessed via https://db.cngb.org/stomics/datasets/STDS0000243. All raw data have been deposited to the CNGB Nucleotide Sequence Archive (https://db.cngb.org/data_resources/project/CNP0004597; https://db.cngb.org/stomics/project/STT0000046) and are publicly available as of the date of publication. Source data are provided with this paper (Supplementary information, Data S1).
