# Supplementary material for: Cochlear nucleus spatial transcriptomes of normal and hearing loss mice reveal a critical role of Spp1 in bushy cells
Source: Cell Res. 2026 Apr 6;36(7):531–50. doi: 10.1038/s41422-026-01246-4 (PMC13287771; doi:10.1038/s41422-026-01246-4)
Supplement: Supplementary file 1 — Supplementary information, Figure S1 [file 41422_2026_1246_MOESM1_ESM.pdf]

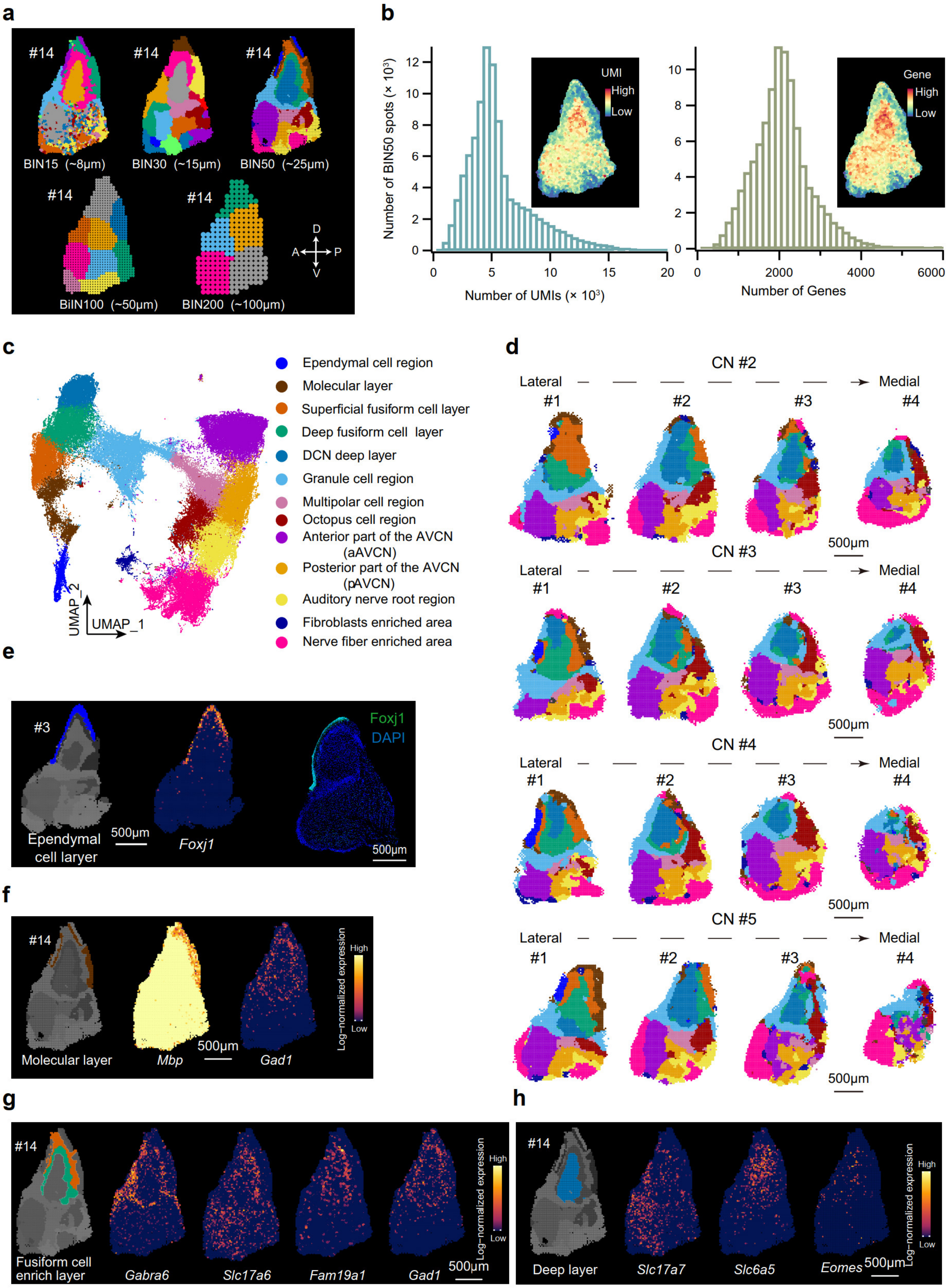

a
#14
BIN15 (~8μm)
BIN30 (~15μm)
BIN50 (~25μm)
BIlN100 (~50μm)
BIN200 (~100μm)
D
A
P
V
b
Number of BIN50 spots (× 10³)
Number of UMIs (× 10³)
UMI
High
Low
Number of Genes
Gene
c
UMAP_1
UMAP_2
Ependymal cell region
Molecular layer
Superficial fusiform cell layer
Deep fusiform cell layer
DCN deep layer
Granule cell region
Multipolar cell region
Octopus cell region
Anterior part of the AVCN (aAVCN)
Posterior part of the AVCN (pAVCN)
Auditory nerve root region
Fibroblasts enriched area
Nerve fiber enriched area
d
CN #2
CN #3
CN #4
CN #5
Lateral
Medial
#1
#2
#3
#4
500μm
e
#3
Ependymal cell laryer
Foxj1
DAPI
500μm
f
#14
Molecular layer
Mbp
Gad1
Log-normalized expression
High
Low
g
Fusiform cell enrich layer
Gabra6
Slc17a6
Fam19a1
Gad1
h
Deep layer
Slc17a7
Slc6a5
Eomes

Supplementary information, Fig. S1: Regional analysis of Stereo-seq data.

a Unsupervised clustering of Stereo-seq data with different BIN sizes.

b The number of captured transcripts (unique molecular identifiers, UMIs) and genes of Stereo-seq data at the resolution of BIN50.

c UMAP visualization of CN subregions from Stereo-seq data at the resolution of BIN50.

d Molecularly defined subregions of the CN in four biological replicates with different starting coordinates.

e Colored ependymal cell layer, spatial expression of *Foxj1* and immunostaining of FOXJ1 in a CN section.

f The DCN molecular region showed a low level of *Mbp* expression.

g Co-expression of *Slc17a6*, *Gabra6*, *Fam19a1* and *Gad1* in the fusiform cell layer.

h The DCN deeper layer contains glutamatergic and glycinergic neurons as indicated by the expression of *Slc6a5*, *Slc17a7* and *Eomes*.
