# Supplementary material for: Cochlear nucleus spatial transcriptomes of normal and hearing loss mice reveal a critical role of Spp1 in bushy cells
Source: Cell Res. 2026 Apr 6;36(7):531–50. doi: 10.1038/s41422-026-01246-4 (PMC13287771; doi:10.1038/s41422-026-01246-4)
Supplement: Supplementary file 2 — Supplementary information, Figure S2 [file 41422_2026_1246_MOESM2_ESM.pdf]

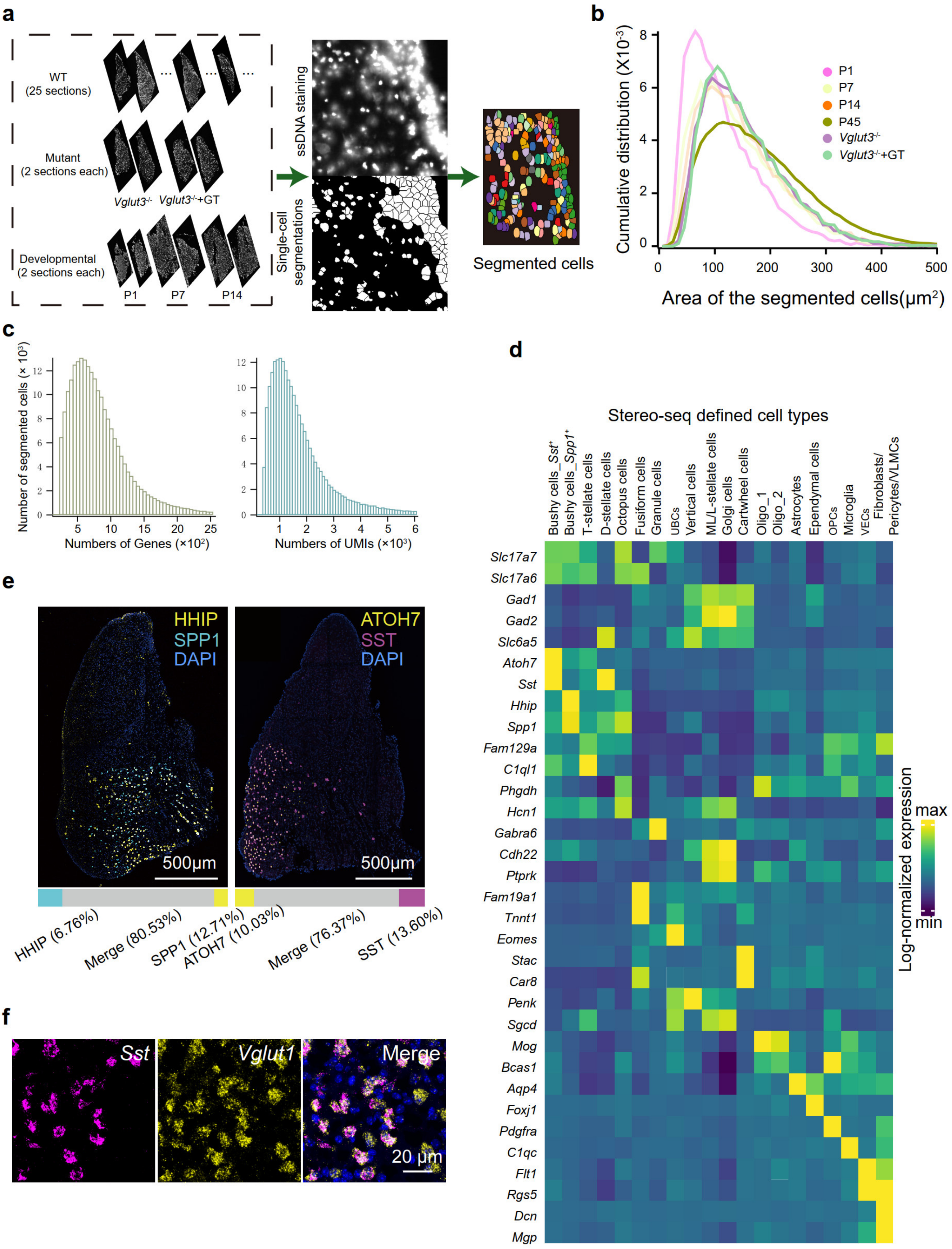
a
WT
(25 sections)
Mutant
(2 sections each)
Vglut3-/-
Vglut3-/-+GT
Developmental
(2 sections each)
P1
P7
P14
ssDNA staining
Single-cell segmentations
Segmented cells
b
Cumulative distribution (X10-3)
Area of the segmented cells(μm2)
P1
P7
P14
P45
Vglut3-/-
Vglut3-/-+GT
c
Number of segmented cells (× 10³)
Numbers of Genes (×10²)
Numbers of UMIs (×10³)
d
Stereo-seq defined cell types
Bushy cells_Sst+
Bushy cells_Spp1+
T-stellate cells
D-stellate cells
Octopus cells
Fusiform cells
Granule cells
UBCs
Vertical cells
ML/L-stellate cells
Golgi cells
Cartwheel cells
Oligo_1
Oligo_2
Astrocytes
Ependymal cells
OPCs
Microglia
VECs
Fibroblasts/
Pericytes/VLMCs
Slc17a7
Slc17a6
Gad1
Gad2
Slc6a5
Atoh7
Sst
Hhip
Spp1
Fam129a
C1ql1
Phgdh
Hcn1
Gabra6
Cdh22
Ptprk
Fam19a1
Tnnt1
Eomes
Stac
Car8
Penk
Sgcd
Mog
Bcas1
Aqp4
Foxj1
Pdgfra
C1qc
Flt1
Rgs5
Dcn
Mgp
max
min
Log-normalized expression
e
HHIP
SPP1
DAPI
ATOH7
SST
DAPI
500μm
500μm
HHIP (6.76%)
Merge (80.53%)
SPP1 (12.71%)
ATOH7 (10.03%)
Merge (76.37%)
SST (13.60%)
f
Sst
Vglut1
Merge
20 μm

Supplementary information, Fig. S2: High resolution single-cell spatial transcriptome of CN.

a Flow chart for the segmentation of cells from stereo-seq data. Outlines are segmented cell boundaries and spatial visualization of segmented cell clusters by unsupervised clustering.

b Distribution of segmented cell area in different mouse groups.

c Distribution of gene and UMI counts of the segmented cells from Stereo-seq data.

d Heatmap showing the expression of marker genes in each Stereo-seq defined cell type.

e Co-immunostaining of SPP1 and HHIP, and SST and ATOH7 in CN sections. Bar graphs show the percentage of single and double labeled cells.

f smFISH revealed co-expression of *Sst* with glutamatergic neurons (*Vglut1*) in the CN.
