# Supplementary material for: Cochlear nucleus spatial transcriptomes of normal and hearing loss mice reveal a critical role of Spp1 in bushy cells
Source: Cell Res. 2026 Apr 6;36(7):531–50. doi: 10.1038/s41422-026-01246-4 (PMC13287771; doi:10.1038/s41422-026-01246-4)
Supplement: Supplementary file 3 — Supplementary information, Figure S3 [file 41422_2026_1246_MOESM3_ESM.pdf]

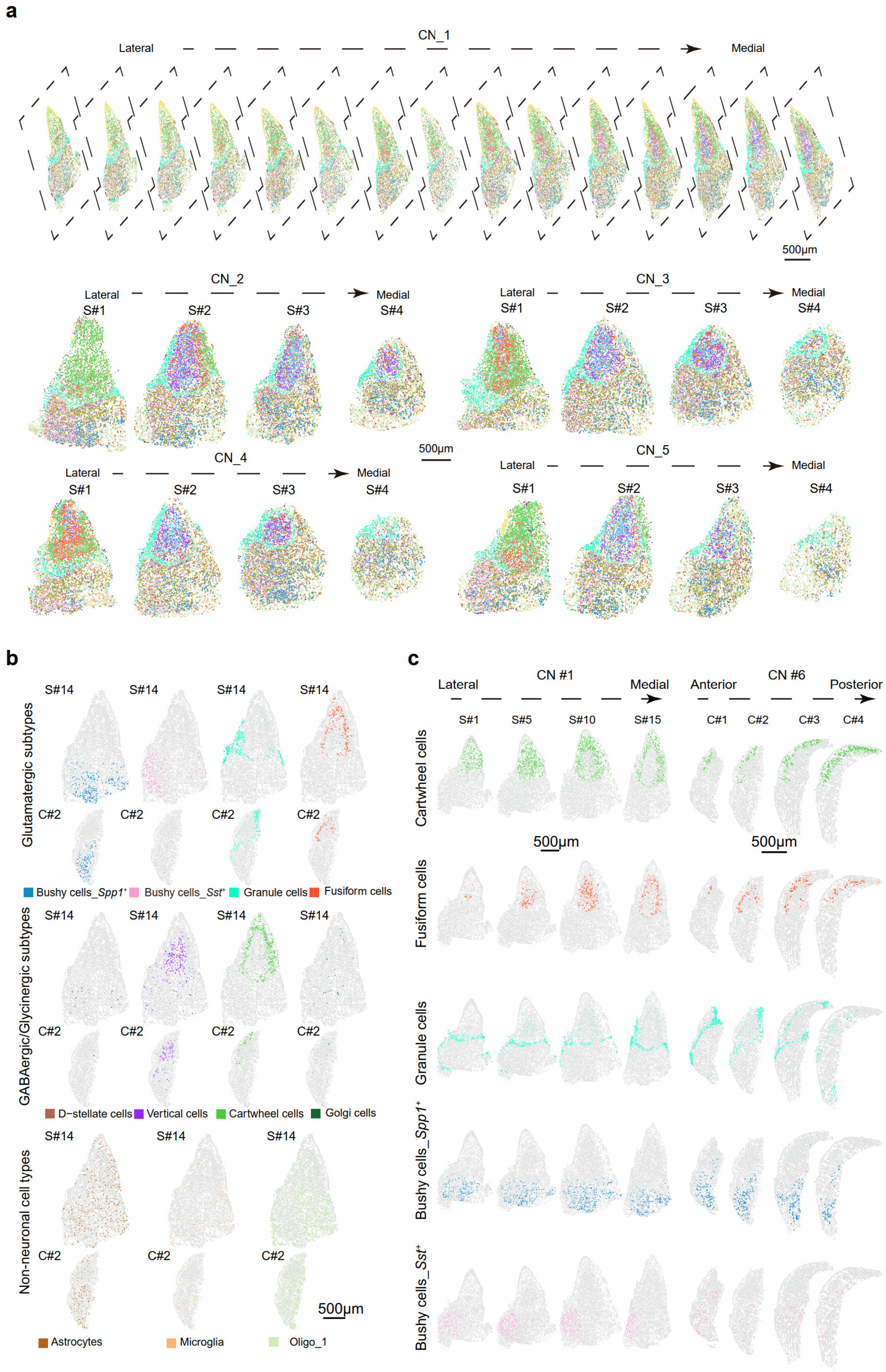

a
CN_1
Lateral
Medial
500μm
CN_2
Lateral
Medial
S#1
S#2
S#3
S#4
CN_3
500μm
CN_4
CN_5
b
Glutamatergic subtypes
S#14
C#2
Bushy cells_Spp1⁺
Bushy cells_Sst⁺
Granule cells
Fusiform cells
GABAergic/Glycinergic subtypes
D−stellate cells
Vertical cells
Cartwheel cells
Golgi cells
Non-neuronal cell types
Astrocytes
Microglia
Oligo_1
500μm
c
CN #1
Lateral
Medial
Anterior
CN #6
Posterior
S#1
S#5
S#10
S#15
C#1
C#2
C#3
C#4
Cartwheel cells
500μm
500μm
Fusiform cells
Granule cells
Bushy cells_Spp1⁺
Bushy cells_Sst⁺

Supplementary information, Fig. S3: Spatial distribution of Stereo-seq defined CN cell types.

a Spatial map of different cell types from 15 consecutive sagittal sections from lateral to medial and 16 biological replicates of sagittal sections from four mice. Cells are colored according to their cell-type identity (Fig. 2a).

b Spatial map of example Stereo-seq defined cell types, including neuronal and non-neuronal types. Indicated cell types are shown in color and other cells are shown in grey. #: numbered sagittal and coronal CN sections.

c Examples of spatial distribution of Stereo-seq defined cell types in different CN sections, and cells are colored according to their annotation.
