# Supplementary material for: Cochlear nucleus spatial transcriptomes of normal and hearing loss mice reveal a critical role of Spp1 in bushy cells
Source: Cell Res. 2026 Apr 6;36(7):531–50. doi: 10.1038/s41422-026-01246-4 (PMC13287771; doi:10.1038/s41422-026-01246-4)
Supplement: Supplementary file 4 — Supplementary information, Figure S4 [file 41422_2026_1246_MOESM4_ESM.pdf]

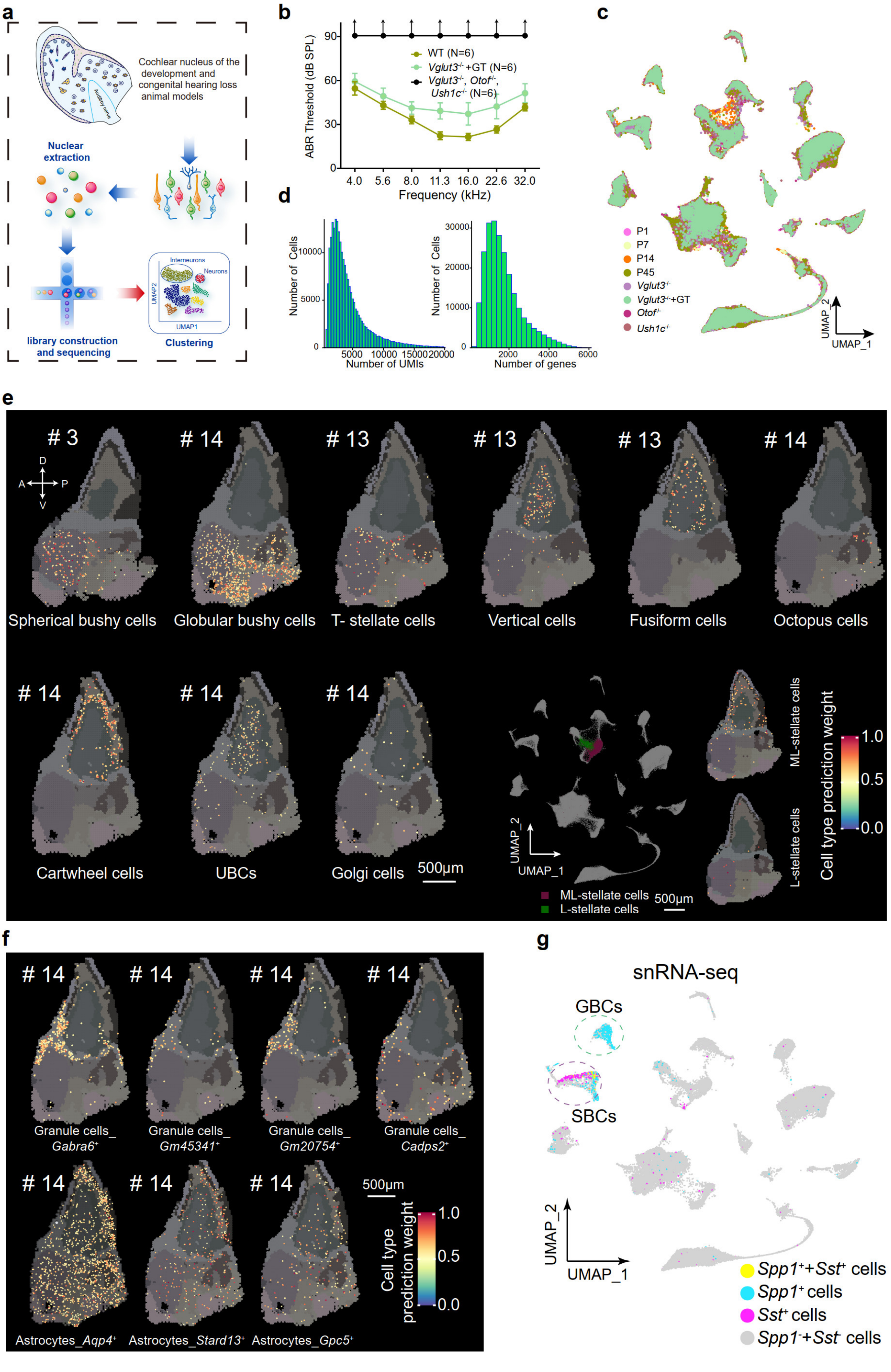

a
Cochlear nucleus of the development and congenital hearing loss animal models
Nuclear extraction
library construction and sequencing
Clustering
Interneurons
Neurons
UMAP1
UMAP2
b
ABR Threshold (dB SPL)
WT (N=6)
Vglut3-/- +GT (N=6)
Vglut3-/-, Otof-/-, Ush1c-/- (N=6)
Frequency (kHz)
4.0 5.6 8.0 11.3 16.0 22.6 32.0
c
P1
P7
P14
P45
Vglut3-/-
Vglut3-/-+GT
Otof-/-
Ush1c-/-
UMAP_2
UMAP_1
d
Number of Cells
Number of UMIs
Number of genes
e
D A P V
Spherical bushy cells
Globular bushy cells
T- stellate cells
Vertical cells
Fusiform cells
Octopus cells
Cartwheel cells
UBCs
Golgi cells
500μm
ML-stellate cells
L-stellate cells
Cell type prediction weight
f
Granule cells_ Gabra6+
Granule cells_ Gm45341+
Granule cells_ Gm20754+
Granule cells_ Cadps2+
Astrocytes_Aqp4+
Astrocytes_Stard13+
Astrocytes_Gpc5+
g
snRNA-seq
GBCs
SBCs
Spp1+ +Sst+ cells
Spp1+ cells
Sst+ cells
Spp1- +Sst cells

Supplementary information, Fig. S4: Profiling of CN SnRNA-seq data and cell type annotation in each group.

a Schematic diagram of snRNA-seq of mouse CN.

b Hearing threshold of normal and mutant mice used for CN snRNA-seq.

c UMAP visualization of cell composition of CN snRNA-seq data from different mouse groups.

d Distribution of gene and UMI counts from the snRNA-seq data.

e Spatial plots showing the location of snRNA-seq defined cell types predicted by RCTD using Stereo-seq data at BIN 20 resolution. UMAP plot showing subclusters of ML/L-stellate cells and their spatial location as predicted by RCTD.

f Spatial localization of snRNA-seq defined astrocyte and granule cell subtypes predicted by RCTD.

g UMAP visualization of the expression of Stereo-seq defined bushy cells markers in snRNA-seq defined two bushy cell subtypes (SBCs and GBCs).
