# Supplementary material for: Cochlear nucleus spatial transcriptomes of normal and hearing loss mice reveal a critical role of Spp1 in bushy cells
Source: Cell Res. 2026 Apr 6;36(7):531–50. doi: 10.1038/s41422-026-01246-4 (PMC13287771; doi:10.1038/s41422-026-01246-4)
Supplement: Supplementary file 5 — Supplementary information, Figure S5 [file 41422_2026_1246_MOESM5_ESM.pdf]

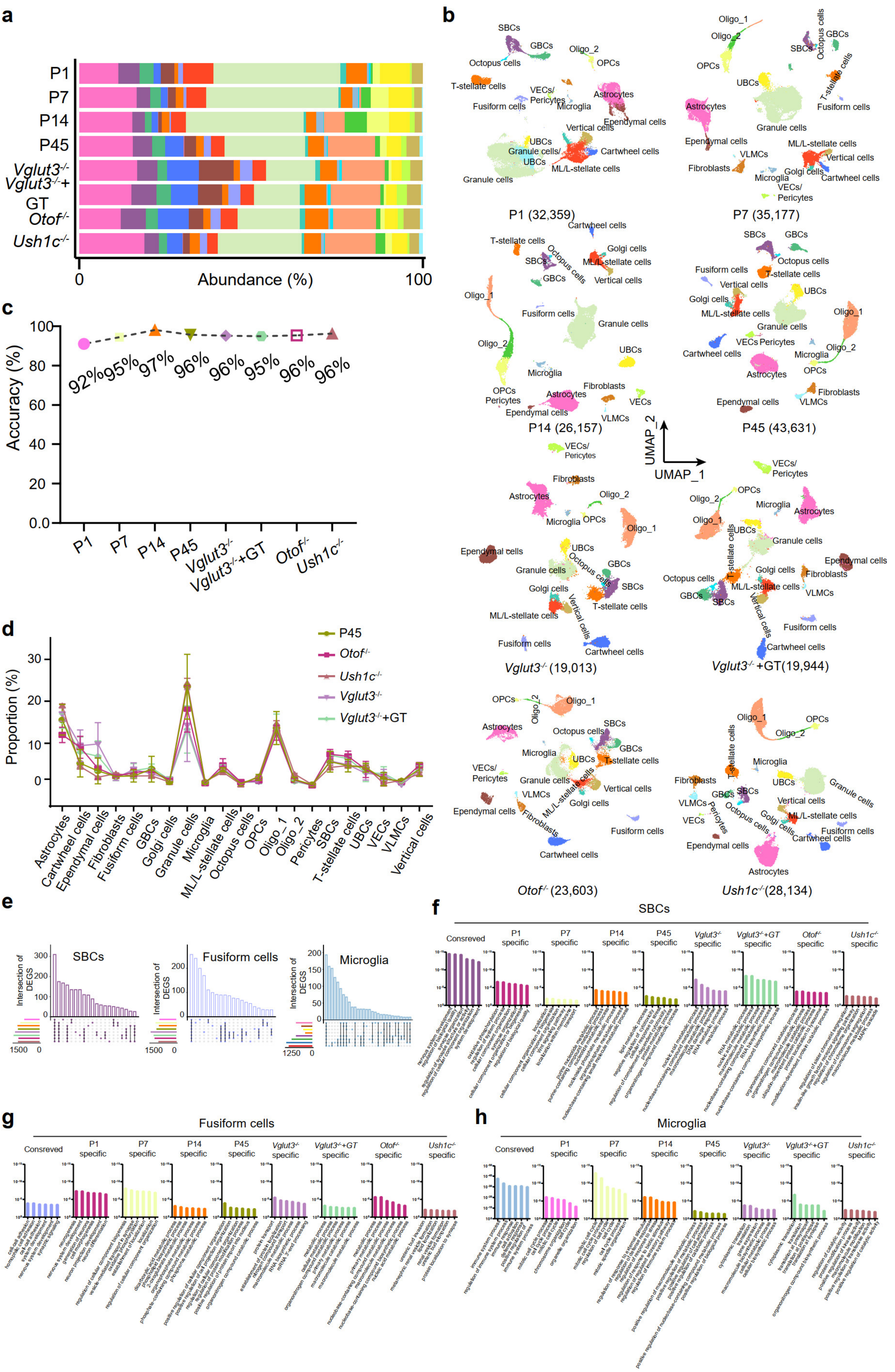
a
P1
P7
P14
P45
Vglut3-/-
Vglut3-/-+GT
Otof-/-
Ush1c-/-
0
Abundance (%)
100
b
P1 (32,359)
P7 (35,177)
P14 (26,157)
P45 (43,631)
Vglut3-/- (19,013)
Vglut3-/- +GT(19,944)
Otof-/- (23,603)
Ush1c-/-(28,134)
UMAP_1
UMAP_2
SBCs
GBCs
Octopus cells
T-stellate cells
Fusiform cells
VECs/ Pericytes
Microglia
Astrocytes
Ependymal cells
Vertical cells
UBCs
Granule cells
Cartwheel cells
ML/L-stellate cells
Golgi cells
Oligo_1
Oligo_2
OPCs
Fibroblasts
VLMCs
VECs
Pericytes
c
Accuracy (%)
100
80
60
40
20
0.0
92%
95%
97%
96%
96%
95%
96%
96%
P1
P7
P14
P45
Vglut3-/-
Vglut3-/-+GT
Otof-/-
Ush1c-/-
d
P45
Otof-/-
Ush1c-/-
Vglut3-/-
Vglut3-/-+GT
Proportion (%)
30
20
10
0
Astrocytes
Cartwheel cells
Ependymal cells
Fibroblasts
Fusiform cells
GBCs
Golgi cells
Granule cells
Microglia
ML/L-stellate cells
Octopus cells
OPCs
Oligo_1
Oligo_2
Pericytes
SBCs
T-stellate cells
UBCs
VECs
VLMCs
Vertical cells
e
SBCs
Fusiform cells
Microglia
Intersection of DEGS
f
SBCs
g
Fusiform cells
h
Microglia
Consreved
P1 specific
P7 specific
P14 specific
P45 specific
Vglut3-/- specific
Vglut3-/-+GT specific
Otof-/- specific
Ush1c-/- specific

Supplementary information, Fig. S5: SnRNA-seq defined cell type annotation and gene expression in each mouse group.

a Bar graphs showing the percentage of CN cells in each group.

b UMAP visualization of cell types identified by snRNA-seq in each mouse group. Cells were colored based on their cell type annotation, and the mixed cell-type was defined by the co-expression of their marker genes

c The dotted line shows the similarity of cell type annotation between the separated and integrated snRNA-seq data.

d No obvious difference was found in the proportion of snRNA-seq defined cell types between the normal and mutant groups. Statistical analysis was performed using one-way ANOVA followed by Bonferroni *post hoc* test.

e-h Upset plots showing the number of shared and divergent marker genes for SBCs, fusiform cells and microglia from snRNA-seq across different mouse groups and GO analysis of these marker genes.
