# Supplementary material for: Cochlear nucleus spatial transcriptomes of normal and hearing loss mice reveal a critical role of Spp1 in bushy cells
Source: Cell Res. 2026 Apr 6;36(7):531–50. doi: 10.1038/s41422-026-01246-4 (PMC13287771; doi:10.1038/s41422-026-01246-4)
Supplement: Supplementary file 6 — Supplementary information, Figure S6 [file 41422_2026_1246_MOESM6_ESM.pdf]

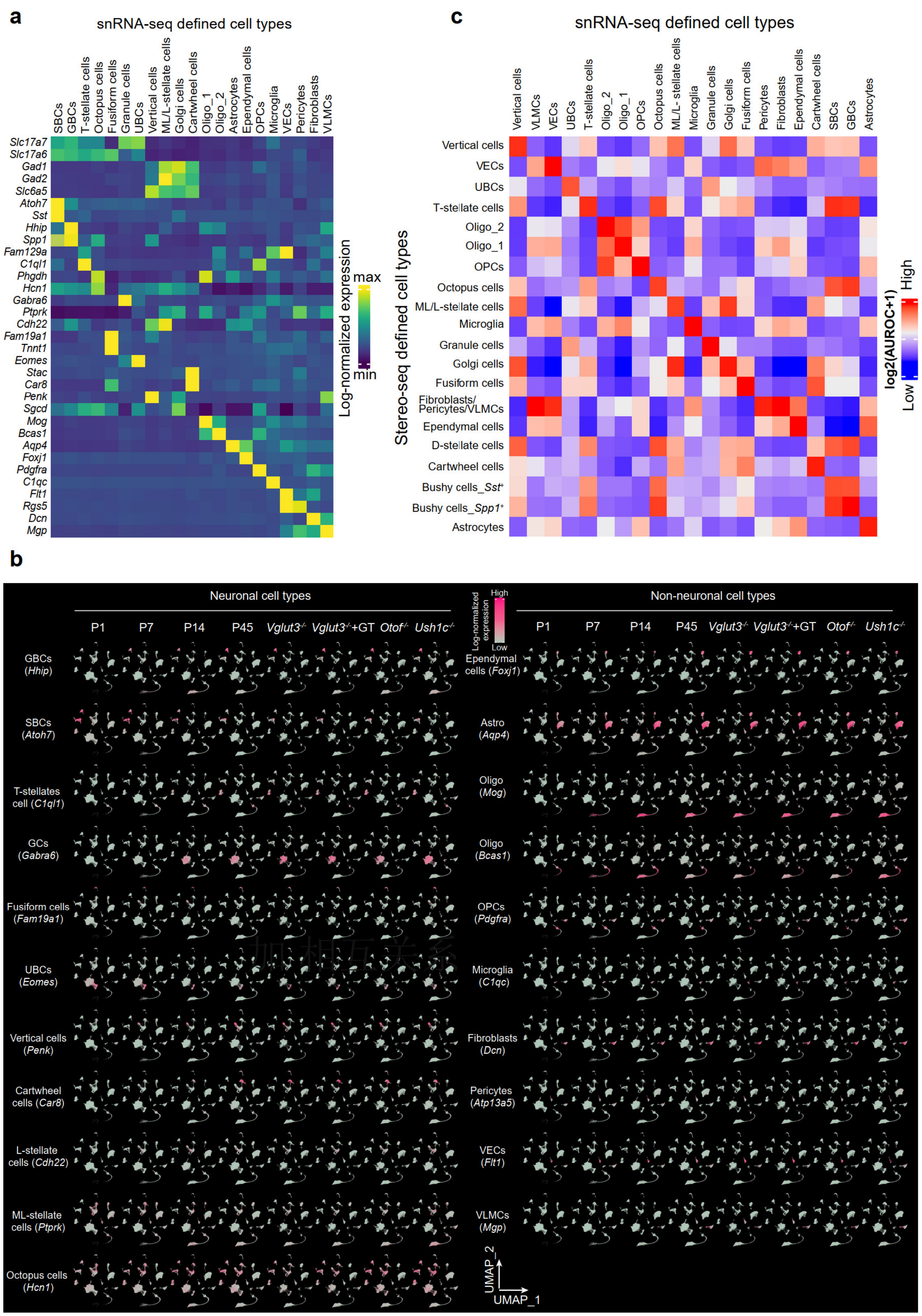
a
snRNA-seq defined cell types
SBCs
GBCs
T-stellate cells
Octopus cells
Fusiform cells
Granule cells
UBCs
Vertical cells
ML/L-stellate cells
Golgi cells
Cartwheel cells
Oligo_1
Oligo_2
Astrocytes
Ependymal cells
OPCs
Microglia
VECs
Pericytes
Fibroblasts
VLMCs
Slc17a7
Slc17a6
Gad1
Gad2
Slc6a5
Atoh7
Sst
Hhip
Spp1
Fam129a
C1ql1
Phgdh
Hcn1
Gabra6
Ptprk
Cdh22
Fam19a1
Tnnt1
Eomes
Stac
Car8
Penk
Sgcd
Mog
Bcas1
Aqp4
Foxj1
Pdgfra
C1qc
Flt1
Rgs5
Dcn
Mgp
Log-normalized expression
max
min
c
snRNA-seq defined cell types
Vertical cells
VLMCs
VECs
UBCs
T-stellate cells
Oligo_2
Oligo_1
OPCs
Octopus cells
ML/L-stellate cells
Microglia
Granule cells
Golgi cells
Fusiform cells
Pericytes
Fibroblasts
Ependymal cells
Cartwheel cells
SBCs
GBCs
Astrocytes
Stereo-seq defined cell types
Vertical cells
VECs
UBCs
T-stellate cells
Oligo_2
Oligo_1
OPCs
Octopus cells
ML/L-stellate cells
Microglia
Granule cells
Golgi cells
Fusiform cells
Fibroblasts/ Pericytes/VLMCs
Ependymal cells
D-stellate cells
Cartwheel cells
Bushy cells_Sst+
Bushy cells_Spp1+
Astrocytes
log2(AUROC+1)
High
Low
b
Neuronal cell types
P1
P7
P14
P45
Vglut3-/-
Vglut3-/-+GT
Otof-/-
Ush1c-/-
GBCs (Hhip)
SBCs (Atoh7)
T-stellates cell (C1ql1)
GCs (Gabra6)
Fusiform cells (Fam19a1)
UBCs (Eomes)
Vertical cells (Penk)
Cartwheel cells (Car8)
L-stellate cells (Cdh22)
ML-stellate cells (Ptprk)
Octopus cells (Hcn1)
Log-normalized expression
High
Low
Non-neuronal cell types
P1
P7
P14
P45
Vglut3-/-
Vglut3-/-+GT
Otof-/-
Ush1c-/-
Ependymal cells (Foxj1)
Astro (Aqp4)
Oligo (Mog)
Oligo (Bcas1)
OPCs (Pdgfra)
Microglia (C1qc)
Fibroblasts (Dcn)
Pericytes (Atp13a5)
VECs (Flt1)
VLMCs (Mgp)
UMAP_2
UMAP_1

Supplementary information, Fig. S6: Marker gene expression and correlation of the averaged profile of gene expression in the two datasets.

a Heatmap showing the expression of marker genes in each Stereo-seq defined cell type.

b UMAP visualization of marker genes in different mouse groups across ages and genotypes.

c Correlation of the major cell types defined by snRNA-seq and Stereo-seq, respectively.
