# Supplementary material for: Cochlear nucleus spatial transcriptomes of normal and hearing loss mice reveal a critical role of Spp1 in bushy cells
Source: Cell Res. 2026 Apr 6;36(7):531–50. doi: 10.1038/s41422-026-01246-4 (PMC13287771; doi:10.1038/s41422-026-01246-4)
Supplement: Supplementary file 7 — Supplementary information, Figure S7 [file 41422_2026_1246_MOESM7_ESM.pdf]

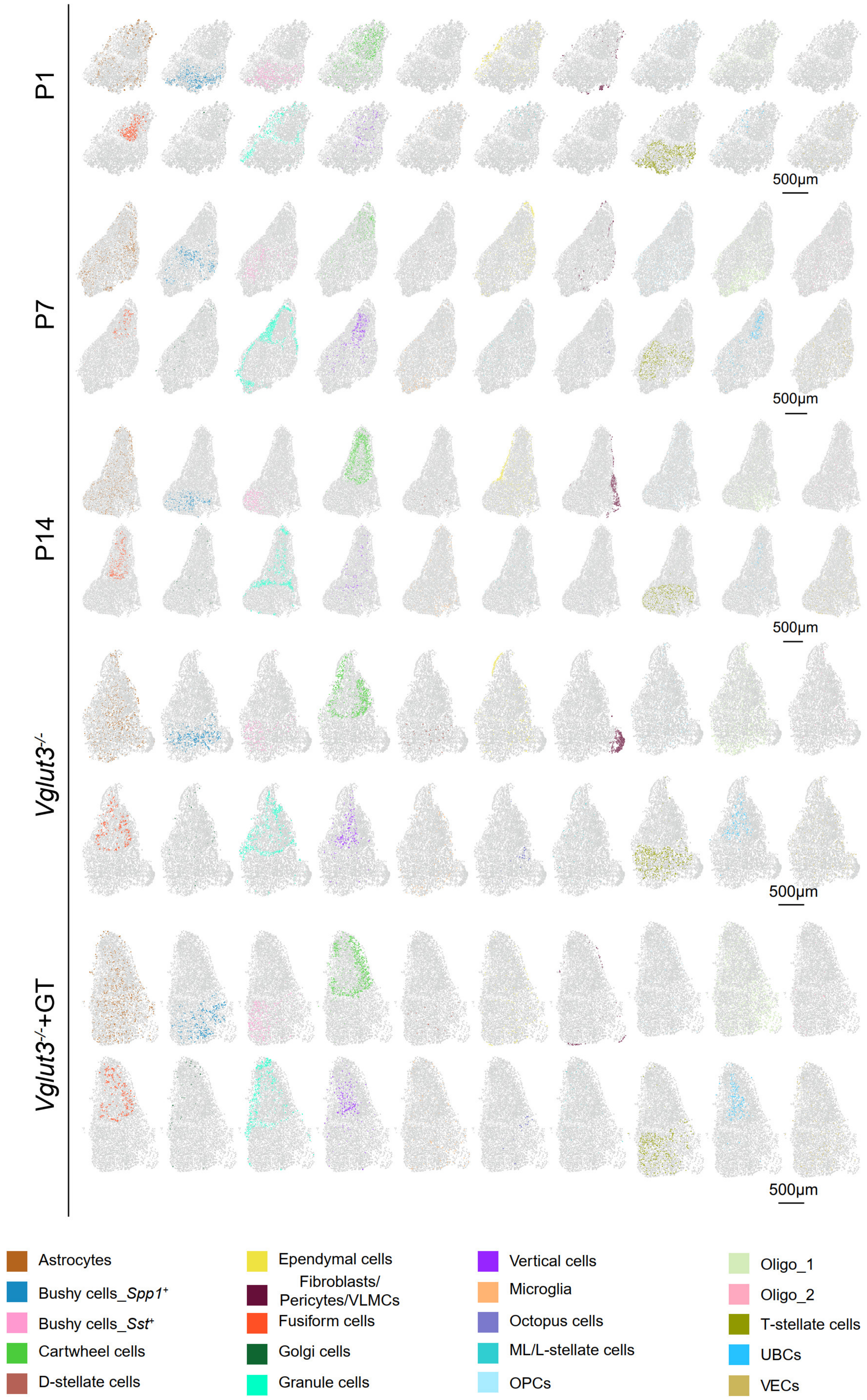

P1
P7
P14
Vglut3-/-
Vglut3-/-+GT
500μm
500μm
500μm
500μm
500μm
Astrocytes
Bushy cells_Spp1+
Bushy cells_Sst+
Cartwheel cells
D-stellate cells
Ependymal cells
Fibroblasts/ Pericytes/VLMCs
Fusiform cells
Golgi cells
Granule cells
Vertical cells
Microglia
Octopus cells
ML/L-stellate cells
OPCs
Oligo_1
Oligo_2
T-stellate cells
UBCs
VECs

Supplementary information, Fig. S7: Spatial cell type distribution of Stereo-seq data in different animal models.

Spatial distribution of different cell types defined by Stereo-seq in P1, P7, P14, *Vglut3*$^{-/-}$ and *Vglut3*$^{-/-}$ +GT mice. OPCs: oligodendrocyte precursor cells, Oligo: oligodendrocytes, VECs: vascular endothelial cells, VLMCs: vascular and leptomeningeal cells, UBCs: unipolar brush cells, A: anterior, P: posterior, V: ventral, D: dorsal.
