# Supplementary material for: Cochlear nucleus spatial transcriptomes of normal and hearing loss mice reveal a critical role of Spp1 in bushy cells
Source: Cell Res. 2026 Apr 6;36(7):531–50. doi: 10.1038/s41422-026-01246-4 (PMC13287771; doi:10.1038/s41422-026-01246-4)
Supplement: Supplementary file 8 — Supplementary information, Figure S8 [file 41422_2026_1246_MOESM8_ESM.pdf]

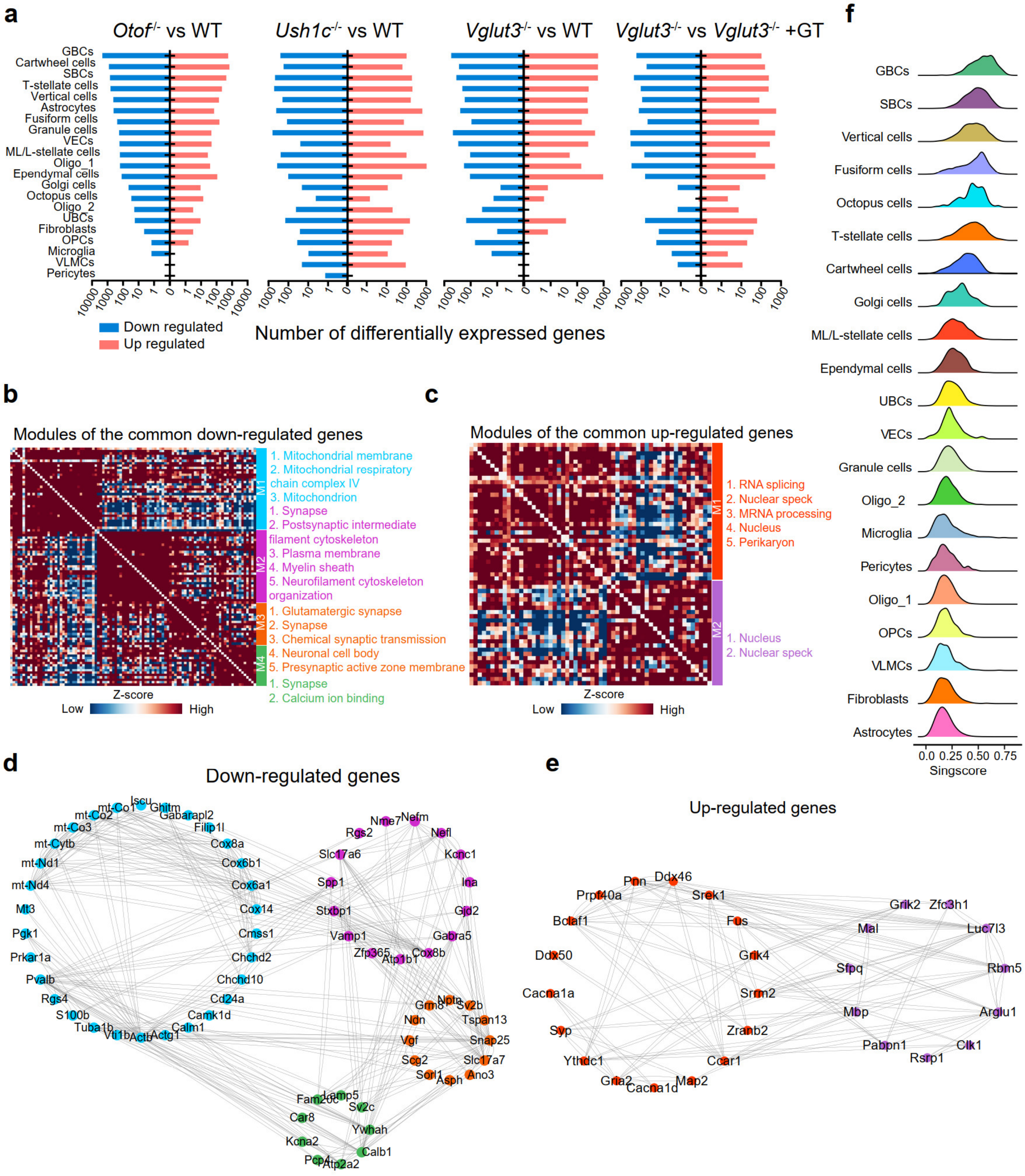
a
Otof-/- vs WT
Ush1c-/- vs WT
Vglut3-/- vs WT
Vglut3-/- vs Vglut3-/- +GT
GBCs
Cartwheel cells
SBCs
T-stellate cells
Vertical cells
Astrocytes
Fusiform cells
Granule cells
VECs
ML/L-stellate cells
Oligo_1
Ependymal cells
Golgi cells
Octopus cells
Oligo_2
UBCs
Fibroblasts
OPCs
Microglia
VLMCs
Pericytes
Down regulated
Up regulated
Number of differentially expressed genes
b
Modules of the common down-regulated genes
1. Mitochondrial membrane
2. Mitochondrial respiratory chain complex IV
3. Mitochondrion
1. Synapse
2. Postsynaptic intermediate filament cytoskeleton
3. Plasma membrane
4. Myelin sheath
5. Neurofilament cytoskeleton organization
1. Glutamatergic synapse
2. Synapse
3. Chemical synaptic transmission
4. Neuronal cell body
5. Presynaptic active zone membrane
1. Synapse
2. Calcium ion binding
M1
M2
M3
M4
Z-score
Low
High
c
Modules of the common up-regulated genes
1. RNA splicing
2. Nuclear speck
3. MRNA processing
4. Nucleus
5. Perikaryon
1. Nucleus
2. Nuclear speck
d
Down-regulated genes
e
Up-regulated genes
f
Singscore
0.0 0.25 0.50 0.75

Supplementary information, Fig. S8: Differential gene expression analysis between wild-type (WT) and hearing loss mouse models based on snRNA-seq data.

a The number of DEGs in snRNA-seq defined major cell types in different comparison groups.

b, c The heat map shows the downregulated and upregulated genes that have been grouped into gene modules. Selected GO terms related to representative modules are shown on the right side.

d, e Protein-protein interaction (PPI) network of the downregulated and upregulated genes of each module. The network was visualized using Cytoscape.

f Ridge plot showing enriched expression of these overlapping DEGs across different snRNA-seq defined cell-types using kernel density curves.
