# Supplementary material for: Cochlear nucleus spatial transcriptomes of normal and hearing loss mice reveal a critical role of Spp1 in bushy cells
Source: Cell Res. 2026 Apr 6;36(7):531–50. doi: 10.1038/s41422-026-01246-4 (PMC13287771; doi:10.1038/s41422-026-01246-4)
Supplement: Supplementary file 9 — Supplementary information, Figure S9 [file 41422_2026_1246_MOESM9_ESM.pdf]

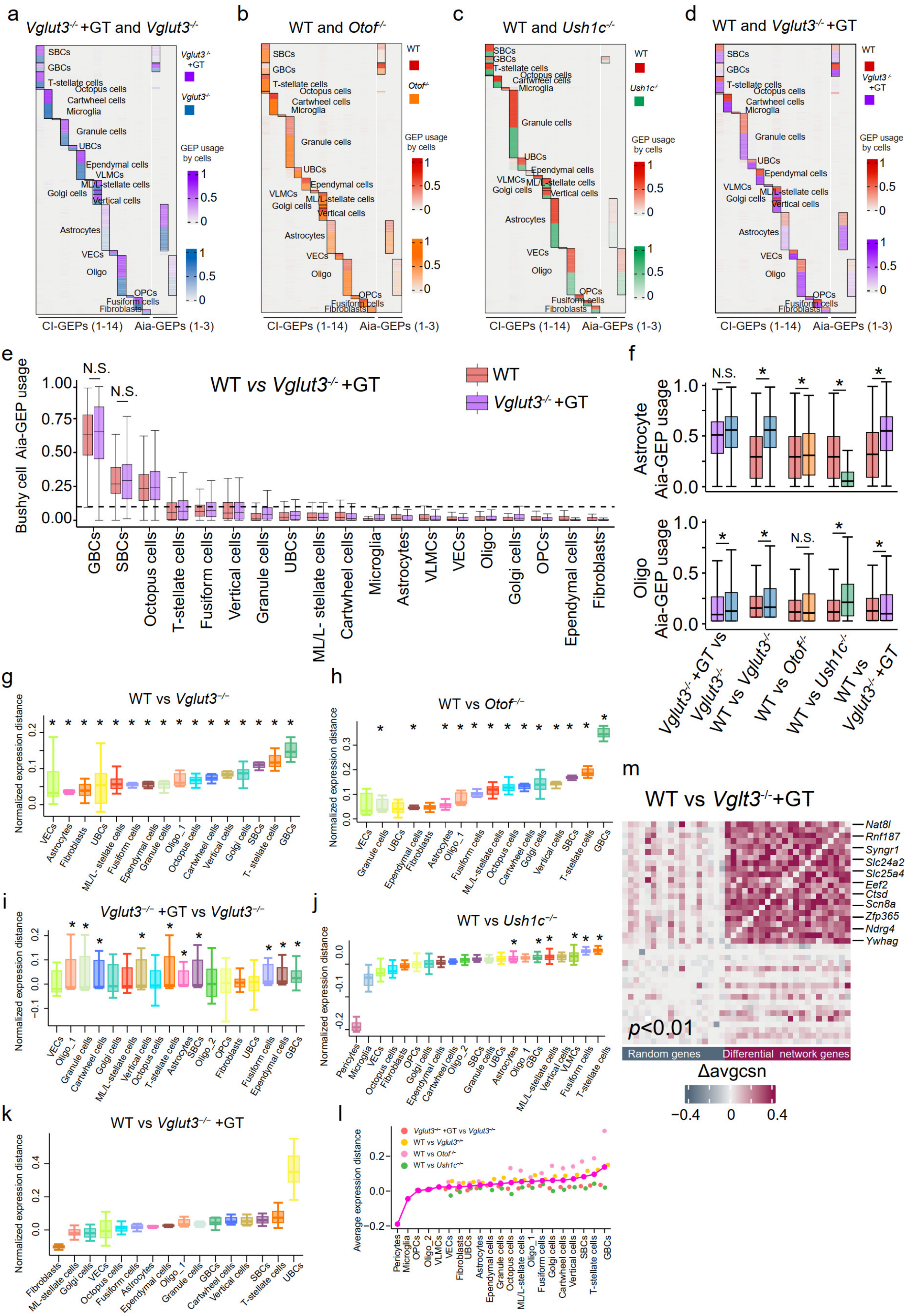
a
Vglut3-/- +GT and Vglut3-/-
b
WT and Otof-/-
c
WT and Ush1c-/-
d
WT and Vglut3-/- +GT
SBCs
GBCs
T-stellate cells
Octopus cells
Cartwheel cells
Microglia
Granule cells
UBCs
Ependymal cells
VLMCs
ML/L-stellate cells
Golgi cells
Vertical cells
Astrocytes
VECs
Oligo
OPCs
Fusiform cells
Fibroblasts
GEP usage by cells
CI-GEPs (1-14)
Aia-GEPs (1-3)
e
WT vs Vglut3-/- +GT
WT
Vglut3-/- +GT
Bushy cell Aia-GEP usage
N.S.
f
Astrocyte Aia-GEP usage
Oligo Aia-GEP usage
Vglut3-/- +GT vs Vglut3-/-
WT vs Vglut3-/-
WT vs Otof-/-
WT vs Ush1c-/-
WT vs Vglut3-/- +GT
g
WT vs Vglut3-/-
h
WT vs Otof-/-
i
Vglut3-/- +GT vs Vglut3-/-
j
WT vs Ush1c-/-
k
WT vs Vglut3-/- +GT
Normalized expression distance
l
Average expression distance
m
WT vs Vglt3-/-+GT
Nat8l
Rnf187
Syngr1
Slc24a2
Slc25a4
Eef2
Ctsd
Scn8a
Zfp365
Ndrg4
Ywhag
p<0.01
Random genes
Differential network genes
Δavgcsn
−0.4
0
0.4

Supplementary information, Fig. S9: Gene expression changes in WT and hearing loss mouse models from snRNA-seq data.

a-d The heatmap shows the percentage usage of GEPs of different mouse models.

e Box and whisker plots showing the percentage usage of Aia-GEPs in different cell types. The dashed line represents the 10% usage of activity programs.

f Box and whisker plots showing the percentage usage of oligodendrocyte and astrocyte Aia-GEPs.

g-l Transcriptional shifts are shown for CN major cell-types in different comparison groups. Box and whisker plots (central line represents the median, boxes represent the interquartile range, and whiskers represent the 5th and 95th quantiles) represent the distribution of normalized pairwise distances, where each observation represents the gene expression shift for a pair of samples from different conditions. Significance levels, estimated by a permutation test (with BH adjustment), are indicated by star symbols on the top. GBCs have the higher averaged expression distance.

m Heatmaps showing the differential network (DN) genes of bushy cells in WT and *Vglut3*$^{-/-}$+GT group using snRNA-seq data.
