# Supplementary material for: Cochlear nucleus spatial transcriptomes of normal and hearing loss mice reveal a critical role of Spp1 in bushy cells
Source: Cell Res. 2026 Apr 6;36(7):531–50. doi: 10.1038/s41422-026-01246-4 (PMC13287771; doi:10.1038/s41422-026-01246-4)
Supplement: Supplementary file 10 — Supplementary information, Figure S10 [file 41422_2026_1246_MOESM10_ESM.pdf]

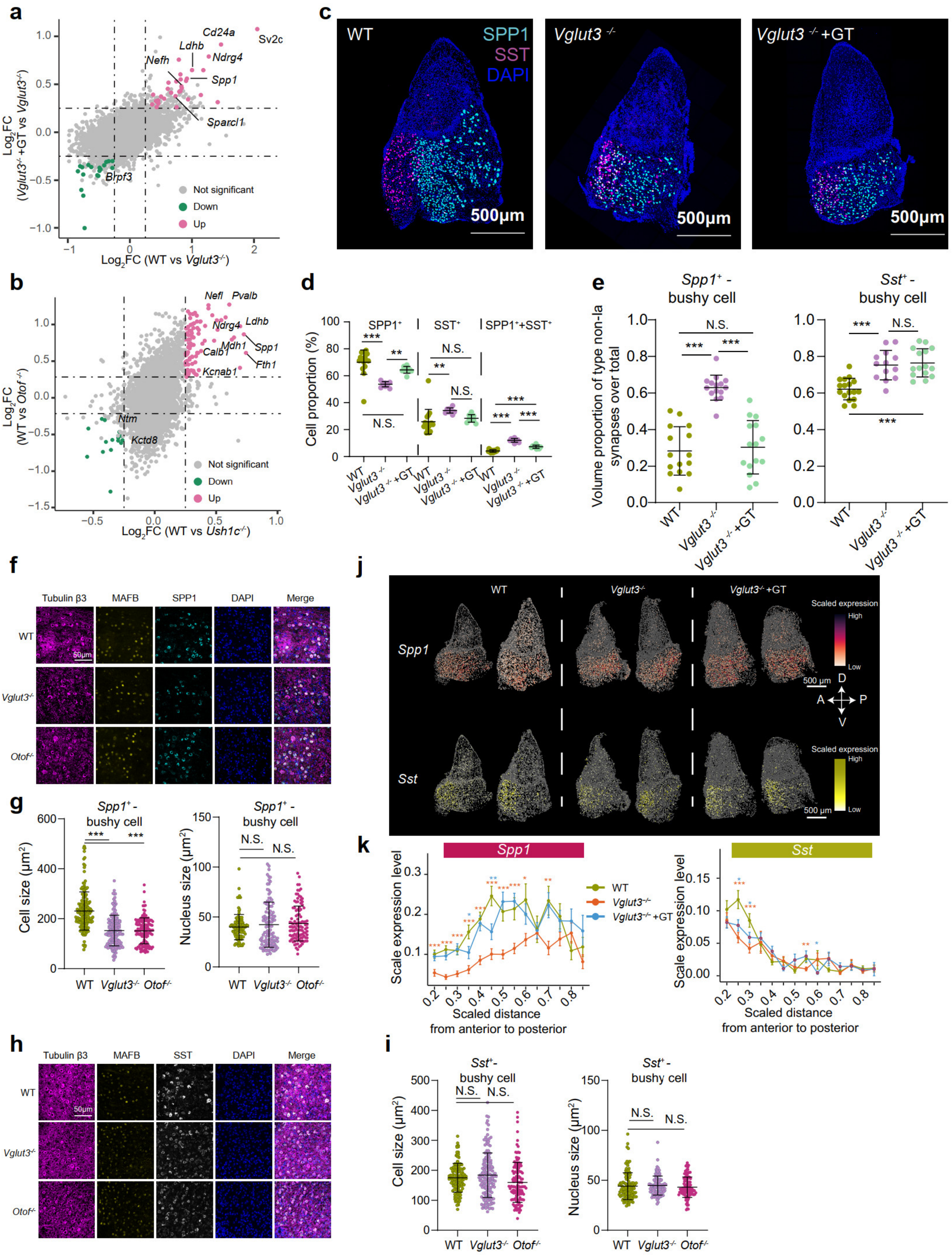

a
Cd24a
Sv2c
Ldhb
Ndrg4
Nefh
Spp1
Sparcl1
Brpf3
Not significant
Down
Up
Log2FC (WT vs Vglut3-/-)
Log2FC (Vglut3-/- +GT vs Vglut3-/-)
b
Nefl
Pvalb
Ndrg4
Ldhb
Mdh1
Spp1
Calb1
Fth1
Kcnab1
Ntm
Kctd8
Not significant
Down
Up
Log2FC (WT vs Ush1c-/-)
Log2FC (WT vs Otof-/-)
c
WT
SPP1
SST
DAPI
Vglut3 -/-
Vglut3 -/- +GT
500μm
d
SPP1+
SST+
SPP1+SST+
N.S.
Cell proportion (%)
WT
Vglut3 -/-
Vglut3 -/- +GT
e
Spp1+ - bushy cell
Sst+ - bushy cell
N.S.
Volume proportion of type non-Ia synapses over total
f
Tubulin β3
MAFB
SPP1
DAPI
Merge
WT
Vglut3-/-
Otof-/-
50μm
g
Spp1+ - bushy cell
Cell size (μm2)
Nucleus size (μm2)
N.S.
h
Tubulin β3
MAFB
SST
DAPI
Merge
i
Sst+- bushy cell
Sst+ - bushy cell
Cell size (μm2)
Nucleus size (μm2)
N.S.
j
WT
Vglut3-/-
Vglut3-/- +GT
Spp1
Sst
Scaled expression
High
Low
500 μm
D
A
P
V
k
Spp1
Sst
WT
Vglut3-/-
Vglut3-/- +GT
Scale expression level
Scaled distance from anterior to posterior

Supplementary information, Fig. S10: Molecular and morphological changes of bushy cells in response to auditory input.

a, b DEGs in *Vglut3*$^{-/-}$ +GT vs *Vglut3*$^{-/-}$, WT vs *Vglut3*$^{-/-}$, WT vs *Ush1c*$^{-/-}$ and WT vs *Otof*$^{-/-}$ of bushy cells from snRNA-seq data.

c, d Immunostaining of cells expressing SPP1 and SST and quantification of their proportions in WT, *Vglut3*$^{-/-}$ and *Vglut3*$^{-/-}$ +GT mice. Statistical analysis was performed using one-way ANOVA followed by Bonferroni *post hoc* test. *$p< 0.05$; **$p < 0.01$; ***$p < 0.001$.

e Quantification of changes in the volume proportion of type non -Ia endbulb of the Held synapse in the two types of bushy cells (Fig. 4d). Statistical analysis was performed using one-way ANOVA followed by Bonferroni *post hoc* test. N.S.: $p > 0.05$; ***$p < 0.001$.

f-i Immunostaining of Tubulin β3 to measure the size of SPP1^{+} - and SST^{+} -bushy cell, showing that the cell size of *Spp1*$^{+}$-bushy cells was significantly reduced in hearing loss mouse models. Statistical analysis was performed using one-way ANOVA followed by Bonferroni *post hoc* test. N.S.: $p > 0.05$; ***$p < 0.001$.

j, k Spatial expression of *Spp1* and *Sst* in different groups (**j**) and their quantification along anterior to posterior (**k**). Statistical analysis was performed using one-way ANOVA followed by Bonferroni *post hoc* test. *$p< 0.05$; **$p < 0.01$; ***$p < 0.001$.
