# Supplementary material for: Cochlear nucleus spatial transcriptomes of normal and hearing loss mice reveal a critical role of Spp1 in bushy cells
Source: Cell Res. 2026 Apr 6;36(7):531–50. doi: 10.1038/s41422-026-01246-4 (PMC13287771; doi:10.1038/s41422-026-01246-4)
Supplement: Supplementary file 11 — Supplementary information, Figure S11 [file 41422_2026_1246_MOESM11_ESM.pdf]

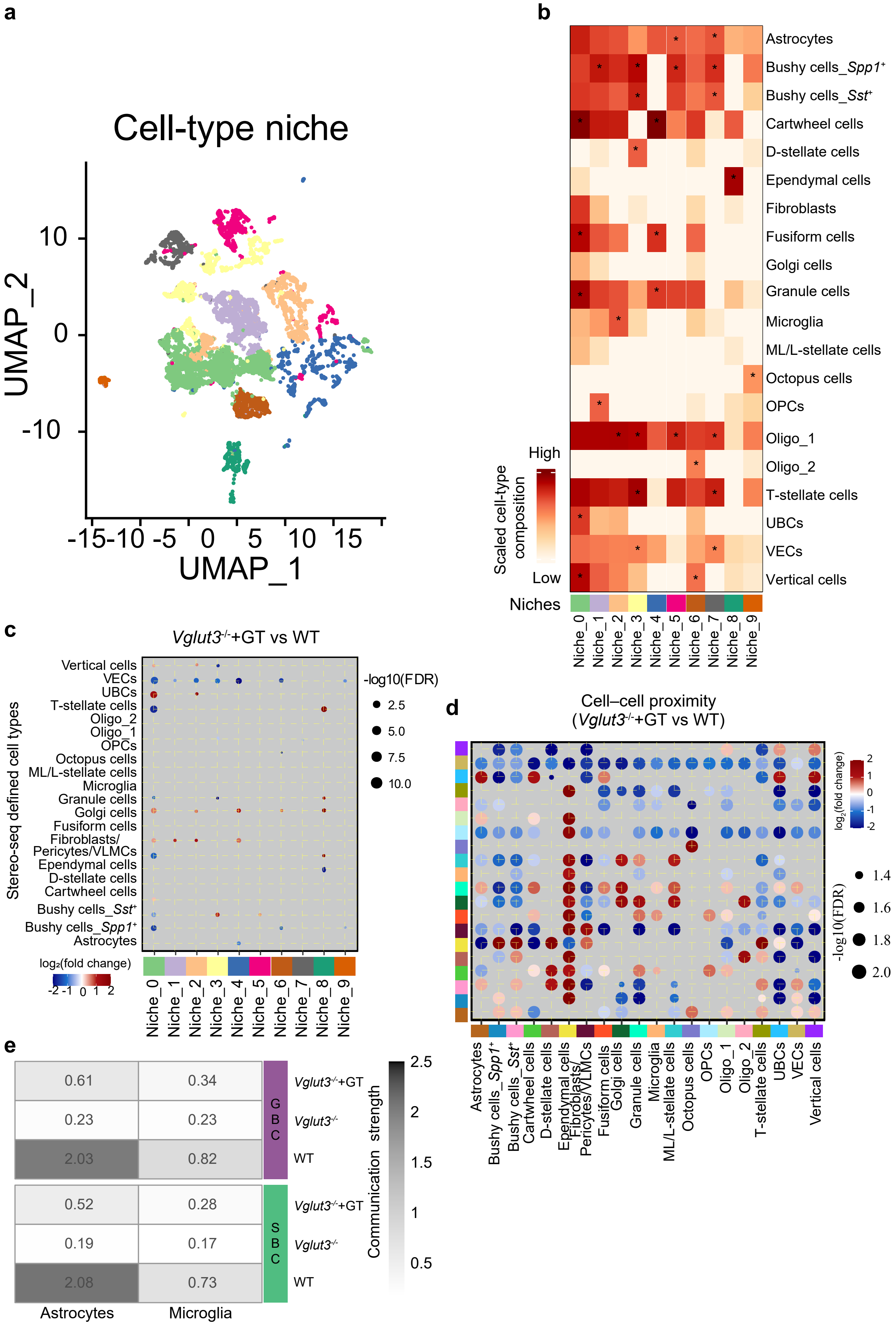
a
Cell-type niche
UMAP_2
UMAP_1
10
0
-10
-15-10 -5 0 5 10 15
b
Astrocytes
Bushy cells_Spp1+
Bushy cells_Sst+
Cartwheel cells
D-stellate cells
Ependymal cells
Fibroblasts
Fusiform cells
Golgi cells
Granule cells
Microglia
ML/L-stellate cells
Octopus cells
OPCs
Oligo_1
Oligo_2
T-stellate cells
UBCs
VECs
Vertical cells
High
Low
Scaled cell-type composition
Niches
Niche_0
Niche_1
Niche_2
Niche_3
Niche_4
Niche_5
Niche_6
Niche_7
Niche_8
Niche_9
c
Vglut3-/-+GT vs WT
Stereo-seq defined cell types
Vertical cells
VECs
UBCs
T-stellate cells
Oligo_2
Oligo_1
OPCs
Octopus cells
ML/L-stellate cells
Microglia
Granule cells
Golgi cells
Fusiform cells
Fibroblasts/
Pericytes/VLMCs
Ependymal cells
D-stellate cells
Cartwheel cells
Bushy cells_Sst+
Bushy cells_Spp1+
Astrocytes
-log10(FDR)
2.5
5.0
7.5
10.0
log2(fold change)
-2 -1 0 1 2
Niche_0
Niche_1
Niche_2
Niche_3
Niche_4
Niche_5
Niche_6
Niche_7
Niche_8
Niche_9
d
Cell–cell proximity
(Vglut3-/-+GT vs WT)
log2(fold change)
2
1
0
-1
-2
-log10(FDR)
1.4
1.6
1.8
2.0
Astrocytes
Bushy cells_Spp1+
Bushy cells_Sst+
Cartwheel cells
D-stellate cells
Ependymal cells
Fibroblasts/
Pericytes/VLMCs
Fusiform cells
Golgi cells
Granule cells
Microglia
ML/L-stellate cells
Octopus cells
OPCs
Oligo_1
Oligo_2
T-stellate cells
UBCs
VECs
Vertical cells
e
0.61 0.34
0.23 0.23
2.03 0.82
GBC
Vglut3-/-+GT
Vglut3-/-
WT
0.52 0.28
0.19 0.17
2.08 0.73
SBC
Vglut3-/-+GT
Vglut3-/-
WT
Astrocytes
Microglia
Communication strength
2.5
2
1.5
1
0.5

Supplementary information, Fig. S11: Changes in the spatial organization of CN cells induced by auditory input

a UMAP of spatial transcriptomic niches based on cell-type composition.

b Scaled median composition of cell types within each niche. Asterisks indicate the main composition of a cell type in one niche compared to other niches.

c The differences in cell type composition in a niche are shown in the dot plot. Statistical analysis was performed using the Wilcoxon rank-sum test.

d Enrichment of cell–cell proximity of different cell types shown in dot plot using Stereo-seq data. Statistical analysis was performed using the Wilcoxon rank-sum test.

e Communication strength between snRNA-seq-defined bushy cells and microglia/astrocytes.
