# Supplementary material for: Cochlear nucleus spatial transcriptomes of normal and hearing loss mice reveal a critical role of Spp1 in bushy cells
Source: Cell Res. 2026 Apr 6;36(7):531–50. doi: 10.1038/s41422-026-01246-4 (PMC13287771; doi:10.1038/s41422-026-01246-4)
Supplement: Supplementary file 12 — Supplementary information, Figure S12 [file 41422_2026_1246_MOESM12_ESM.pdf]

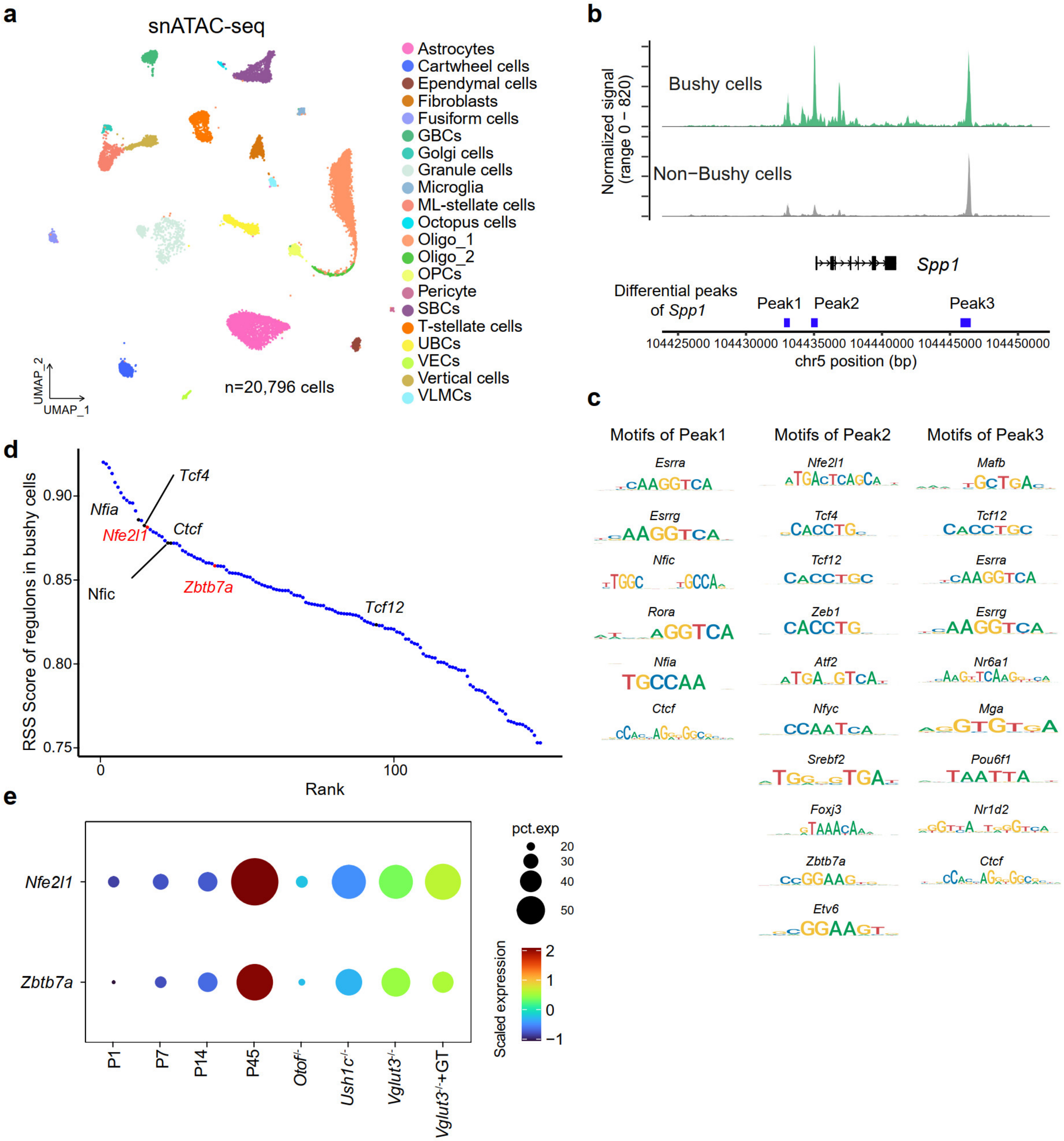
a
snATAC-seq
Astrocytes
Cartwheel cells
Ependymal cells
Fibroblasts
Fusiform cells
GBCs
Golgi cells
Granule cells
Microglia
ML-stellate cells
Octopus cells
Oligo_1
Oligo_2
OPCs
Pericyte
SBCs
T-stellate cells
UBCs
VECs
Vertical cells
VLMCs
UMAP_2
UMAP_1
n=20,796 cells
b
Normalized signal (range 0 - 820)
Bushy cells
Non−Bushy cells
Spp1
Differential peaks of Spp1
Peak1
Peak2
Peak3
104425000 104430000 104435000 104440000 104445000 104450000
chr5 position (bp)
c
Motifs of Peak1
Esrra
Esrrg
Nfic
Rora
Nfia
Ctcf
Motifs of Peak2
Nfe2l1
Tcf4
Tcf12
Zeb1
Atf2
Nfyc
Srebf2
Foxj3
Zbtb7a
Etv6
Motifs of Peak3
Mafb
Tcf12
Esrra
Esrrg
Nr6a1
Mga
Pou6f1
Nr1d2
Ctcf
d
RSS Score of regulons in bushy cells
0.90
0.85
0.80
0.75
Tcf4
Nfia
Nfe2l1
Ctcf
Nfic
Zbtb7a
Tcf12
0
100
Rank
e
Nfe2l1
Zbtb7a
P1
P7
P14
P45
Otof-/-
Ush1c-/-
Vglut3-/-
Vglut3-/-+GT
pct.exp
20
30
40
50
Scaled expression
2
1
0
−1

Supplementary information, Fig. S12: Single-nucleus analysis of chromatin accessibility and expression of *Spp1*-related transcription factors across experimental groups.

a UMAP projection of CN snATAC-seq data at P45, colored by annotated cell types.

b Differential chromatin accessibility peaks near the *Spp1* locus between bushy cells and non-bushy cells, as identified by snATAC-seq.

c Motif enrichment within *Spp1*-associated differential peaks (Peak1–3), analyzed using HOMER with a hypergeometric test.

d The Regulon Specificity Score (RSS) was derived from the SCENIC analysis of snRNA-seq data in bushy cells. The red-marked TFs were predicted to regulate *Spp1* expression base on JASPAR2020 dataset.

e Dot plot showing expression levels of *Nfe2l1* and *Zbtb7a* in bushy cells across experimental groups, based on snRNA-seq data.
