# Supplementary material for: Cochlear nucleus spatial transcriptomes of normal and hearing loss mice reveal a critical role of Spp1 in bushy cells
Source: Cell Res. 2026 Apr 6;36(7):531–50. doi: 10.1038/s41422-026-01246-4 (PMC13287771; doi:10.1038/s41422-026-01246-4)
Supplement: Supplementary file 13 — Supplementary information, Figure S13 [file 41422_2026_1246_MOESM13_ESM.pdf]

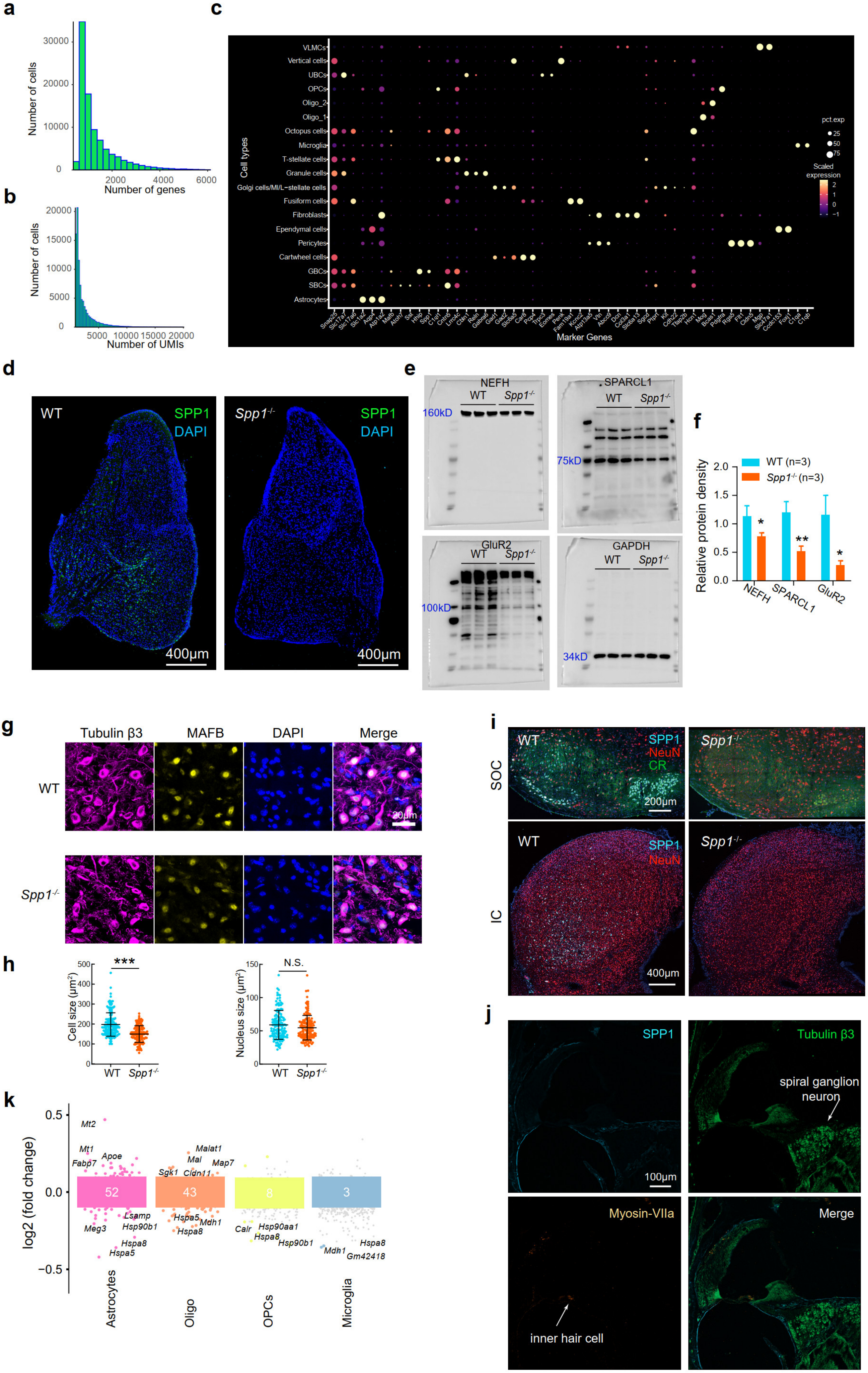
a
Number of cells
Number of genes
b
Number of cells
Number of UMIs
c
Cell types
Marker Genes
pct.exp
Scaled expression
VLMCs
Vertical cells
UBCs
OPCs
Oligo_2
Oligo_1
Octopus cells
Microglia
T-stellate cells
Granule cells
Golgi cells/MI/L-stellate cells
Fusiform cells
Fibroblasts
Ependymal cells
Pericytes
Cartwheel cells
GBCs
SBCs
Astrocytes
d
WT
SPP1
DAPI
Spp1-/-
400μm
e
NEFH
SPARCL1
GluR2
GAPDH
160kD
75kD
100kD
34kD
f
Relative protein density
WT (n=3)
Spp1-/- (n=3)
NEFH
SPARCL1
GluR2
g
Tubulin β3
MAFB
DAPI
Merge
20μm
h
Cell size (μm²)
Nucleus size (μm²)
N.S.
i
SOC
IC
NeuN
CR
200μm
j
Myosin-VIIa
spiral ganglion neuron
inner hair cell
100μm
k
log2 (fold change)
Astrocytes
Oligo
OPCs
Microglia

Supplementary information, Fig. S13: Molecular and morphology changes of CN cell types in *Spp1*$^{-/-}$ mice.

a, b Distribution of gene and UMI counts of snRNA-seq data from WT and *Spp1*$^{-/-}$ mice.

c Dot plot of marker gene expression in each cell type identified by snRNA-seq from WT and *Spp1*$^{-/-}$ mice.

d Immunostaining of SPP1 and no SPP1 expression was found in CN of *Spp1*$^{-/-}$ mice.

e, f Western blotting analysis of the expression of NEFH, SPARCL1 and GluR2 in CN tissue and their expression level was reduced in *Spp1*$^{-/-}$ mice. Protein bands were captured sequentially on a membrane. Statistical analysis was performed by two-tailed unpaired Student's *t* test. *p< 0.05 and **p< 0.01.

g, h Immunofluorescence staining to measure the soma size of bushy cells showed that soma size was significantly reduced in *Spp1*$^{-/-}$ mice. Statistical analysis was performed using the Mann-Whitney *U* test. N.S.: $p > 0.05$ and ***$p < 0.001$.

i, j SPP1 expression in the superior olivary complex (SOC), inferior colliculus (IC), inner hair cells and spiral ganglion neurons by immunostaining.
