# Supplementary material for: Cochlear nucleus spatial transcriptomes of normal and hearing loss mice reveal a critical role of Spp1 in bushy cells
Source: Cell Res. 2026 Apr 6;36(7):531–50. doi: 10.1038/s41422-026-01246-4 (PMC13287771; doi:10.1038/s41422-026-01246-4)
Supplement: Supplementary file 14 — Supplementary information, Figure S14 [file 41422_2026_1246_MOESM14_ESM.pdf]

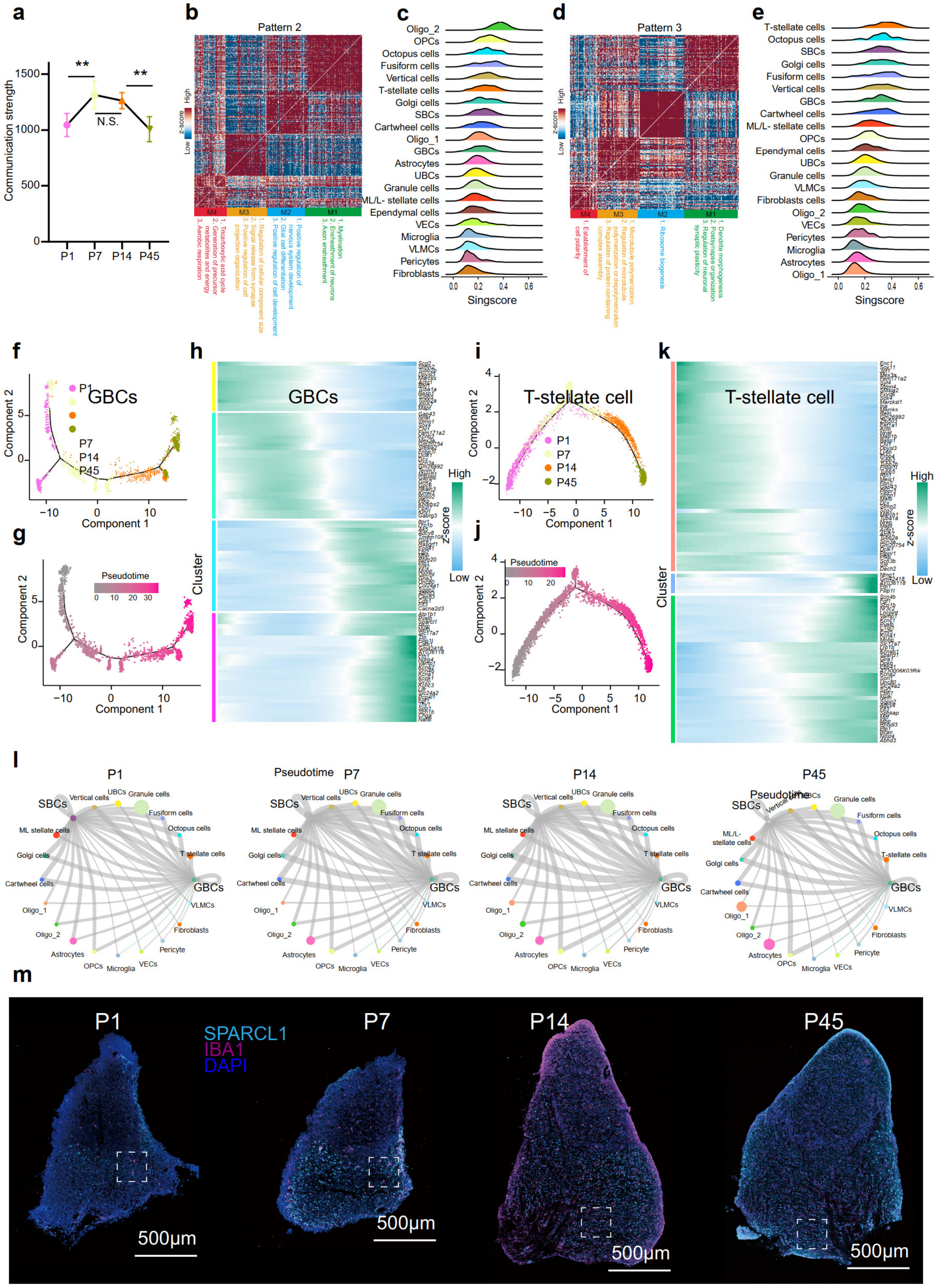

a
Communication strength
1500
1000
500
0
**
N.S.
**
P1
P7
P14
P45
b
Pattern 2
z-score
High
Low
M4
M3
M2
M1
1. Tricarboxylic acid cycle
2. Generation of precursor metabolites and energy
3. Aerobic respiration
1. Regulation of cellular component size
2. Signal release from synapse
3. Positive regulation of cell projection organization
1. Positive regulation of nervous system development
2. Glial cell differentiation
3. Positive regulation of cell development
1. Myelination
2. Ensheathment of neurons
3. Axon ensheathment
c
Oligo_2
OPCs
Octopus cells
Fusiform cells
Vertical cells
T-stellate cells
Golgi cells
SBCs
Cartwheel cells
Oligo_1
GBCs
Astrocytes
UBCs
Granule cells
ML/L- stellate cells
Ependymal cells
VECs
Microglia
VLMCs
Pericytes
Fibroblasts
0.0
0.2
0.4
0.6
Singscore
d
Pattern 3
z-score
High
Low
M4
M3
M2
M1
1. Establishment of cell polarity
1. Microtubule polymerization
2. Regulation of microtubule polymerization or depolymerization
3. Regulation of protein-containing complex assembly
1. Ribosome biogenesis
1. Dendrite morphogenesis
2. Postsynapse organization
3. Regulation of neuronal synaptic plasticity
e
T-stellate cells
Octopus cells
SBCs
Golgi cells
Fusiform cells
Vertical cells
GBCs
Cartwheel cells
ML/L- stellate cells
OPCs
Ependymal cells
UBCs
Granule cells
VLMCs
Fibroblasts cells
Oligo_2
VECs
Pericytes
Microglia
Astrocytes
Oligo_1
0.0
0.2
0.4
0.6
Singscore
f
GBCs
P1
P7
P14
P45
Component 2
Component 1
g
Pseudotime
0
10
20
30
Component 2
Component 1
h
GBCs
Cluster
High
Low
z-score
i
T-stellate cell
P1
P7
P14
P45
Component 2
Component 1
j
Pseudotime
0
10
20
Component 2
Component 1
k
T-stellate cell
Cluster
High
Low
z-score
l
P1
P7
P14
P45
Pseudotime
SBCs
Vertical cells
UBCs
Granule cells
Fusiform cells
ML stellate cells
ML/L- stellate cells
Octopus cells
Golgi cells
T stellate cells
T-stellate cells
Cartwheel cells
GBCs
Oligo_1
VLMCs
Oligo_2
Fibroblasts
Astrocytes
Pericyte
OPCs
Microglia
VECs
m
P1
P7
P14
P45
SPARCL1
IBA1
DAPI
500μm

Supplementary information, Fig. S14: Dynamic changes of gene expression during CN development.

a Changes in communication strength during development. Statistical analysis was performed by two-tailed unpaired Student's *t* test. $^{**}p < 0.01$.

b-d Heatmap showing the gene modules of genes from pattern 2 (**a**) and 3 (**c**), respectively. Selected GO terms related to representative modules are shown at the bottom. Ridge plot showing enriched expression of the pattern 2 (**b**) and 3 (**d**) gene sets across different snRNA-seq defined cell-types using kernel density curves.

f-k Pseudo-time trajectories of GBCs and T-stellate cells at different developmental stages. Gene expression patterns over pseudo-time in the two cell types are listed.

l Cell-chat networks of the snRNA-seq defined GBCs and SBCs with other cell types at different developmental stages, with the bandwidth representing the communication strength.

m Co-immunostaining of SPARCL1 and IBA1in CN sections. We used SPARCL1 to label *Spp1*$^{+}$-bushy cells because SPP1 cannot be stained due to the low expression of *Spp1* at P1 and P7 (related to Fig. 7j).
